# High-Strain-Induced Local Modification of the Electronic Properties of VO_2_ Thin Films

**DOI:** 10.1021/acsaelm.2c01176

**Published:** 2022-11-18

**Authors:** Yorick A. Birkhölzer, Kai Sotthewes, Nicolas Gauquelin, Lars Riekehr, Daen Jannis, Emma van der Minne, Yibin Bu, Johan Verbeeck, Harold J. W. Zandvliet, Gertjan Koster, Guus Rijnders

**Affiliations:** † MESA+ Institute of Nanotechnology, University of Twente, P.O. Box 217, 7500AEEnschede, The Netherlands; ¶ Electron Microscopy for Materials Science (EMAT), University of Antwerp, Groenenborgerlaan 171, 2020Antwerp, Belgium

**Keywords:** C-AFM, VO_2_, pressure, nanoindentation, metal−insulator transition, nanoscale transport spectroscopy, phase diagram

## Abstract

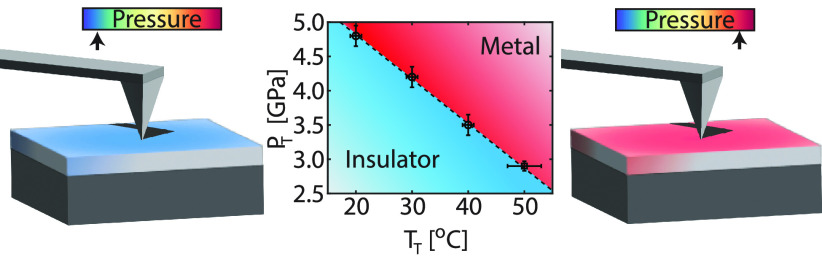

Vanadium dioxide (VO_2_) is a popular candidate for electronic and optical switching applications due to its well-known semiconductor–metal transition. Its study is notoriously challenging due to the interplay of long- and short-range elastic distortions, as well as the symmetry change and the electronic structure changes. The inherent coupling of lattice and electronic degrees of freedom opens the avenue toward mechanical actuation of single domains. In this work, we show that we can manipulate and monitor the reversible semiconductor-to-metal transition of VO_2_ while applying a controlled amount of mechanical pressure by a nanosized metallic probe using an atomic force microscope. At a critical pressure, we can reversibly actuate the phase transition with a large modulation of the conductivity. Direct tunneling through the VO_2_–metal contact is observed as the main charge carrier injection mechanism before and after the phase transition of VO_2_. The tunneling barrier is formed by a very thin but persistently insulating surface layer of the VO_2_. The necessary pressure to induce the transition decreases with temperature. In addition, we measured the phase coexistence line in a hitherto unexplored regime. Our study provides valuable information on pressure-induced electronic modifications of the VO_2_ properties, as well as on nanoscale metal-oxide contacts, which can help in the future design of oxide electronics.

## 1 Introduction

Electronic phase transitions play a key role in the study of quantum materials and promise versatile applications in the next generation of low-power electronic devices for information processing and storage,^1^ fast optoelectronic switches,^2^ actuators,^3^ filters,^4^ hydrogen storage,^5^ and smart windows.^6^ With a phase transition temperature close to room temperature and an electrical resistance change of several orders of magnitude,^7^ vanadium dioxide (VO_2_) is one of the best-studied candidates from the class of transition metal oxides.

The reversible semiconductor-to-metal transition, often referred to as metal–insulator transition (MIT), can be triggered by various stimuli, such as heat,^8^ light,^9^ mechanical pressure,^10^ and electric^11^ and magnetic fields,^12^ which opens the possibility for realizing switching devices between well-defined off (insulating) and on (metallic) states. In order to better understand the microscopic origin of the MIT, VO_2_ has been investigated with state-of-the-art (pump–probe) spectroscopy techniques employing radiation all across the electromagnetic spectrum from hard and soft X-rays,^13^ to XUV,^14^ infrared,^15^ THz,^16^ microwave,^17^ and radio frequency,^18^ as well as electron microscopy.^19,20^ The interested reader is referred to the review by Shao et al.^21^ Our current understanding is that the electronic phase transition of VO_2_ is inherently coupled to an accompanying structural phase transition from a monoclinic crystal structure at low temperature to a tetragonal structure at high temperature. The monoclinic phase is defined by a characteristic dimerization and tilt motif of the vanadium ions, whereas the tetragonal phase is isostructural to rutile TiO_2_. However, despite decades of research, the exact mechanism of the phase transition (Mott,^22^ Peierls,^23^ order–disorder^24^) is still under debate.

In this context, the possibility of switching thin film quantum materials by pressure has so far received limited attention. In the case of VO_2_, an externally applied pressure has been used before to induce the MIT.^25−30^ In the work of Park et al.,^27^ VO_2_ single-crystal nanobeams were studied under the influence of uniaxial stress in a scanning electron microscope. From these measurements, a pressure–temperature (*P*–*T*) phase diagram was constructed. To date, the MIT was controlled on a macroscopic scale or by a macroscopic probe, but reliable and reversible nanoscale control of the material and its properties remained elusive. We expect to gain important insights provided that sufficient pressure levels are achieved, and pressure is directly applied in the crystallographic direction where the dimerization takes place.

There are two main routes to introduce strain or pressure into the system. One is strain engineering such as heteroepitaxy, where a thin film is grown on a crystalline substrate that acts as a template and clamps the in-plane lattice parameter. Epitaxial strain can be anisotropic but is usually biaxial, i.e., applying pressure within the interfacial plane and only indirectly—through the Poisson effect—in the direction of the surface normal. This is in contrast to the second route in which pressure is applied externally by, for instance, a diamond anvil cell (DAC).^28^ Thanks to their limited contact area in the range of a few hundred μm in diameter, DACs can reach uniaxial pressures on the order of hundreds of GPa. In a DAC, a pressure medium can be used to convert uniaxial pressure into hydrostatic, isotropic pressure.

Here, we combine the two routes, (i) the effect of epitaxy, i.e., in-plane clamping, with (ii) the principle of a uniaxial DAC, albeit on an approximately 4 orders of magnitude smaller length scale by using a diamond AFM tip. As we will show, this allows manipulation and symmetry breaking of the material under study. Atomic force microscopy (AFM) is a very suitable method for applying large pressures (up to tens of GPa) on various types of materials. To date, nanoscale manipulation with an AFM has been performed on NiO,^31^ Sr_2_IrO_4_,^32^ SrTiO_3_,^33^ V_2_O_3_,^34^ VO_2_,^35^ and two-dimensional ice.^36^ In this way, the material can locally be manipulated while, for instance, the transport mechanism can be determined simultaneously from nanoscale current–voltage (*I*(*V*)) spectroscopy. For more background on nanoelectrical AFM measurements, the interested reader is referred to the books by Celano^37^ and Lanza.^38^ Schrecongost et al.^35^ were able to demonstrate (irreversible) local manipulations of VO_2_ under very large electric fields. By applying probe-induced strain or by a highly biased AFM probe, a persistent metallic phase or an insulating phase could be created through plastic deformation and electrochemical modification.

In the present study, we apply an external, local, and predominantly uniaxial stress on an epitaxially grown and biaxially prestrained VO_2_ thin film using a conductive AFM tip in order to probe the reversible MIT in a fully elastic regime without changing the stoichiometry. Furthermore, we elucidate the electrical contact mechanism and expand the *P*–*T* phase diagram by an order of magnitude with respect to the seminal work of Park et al.^27^

## 2 Results and Discussion

**Figure 1:**
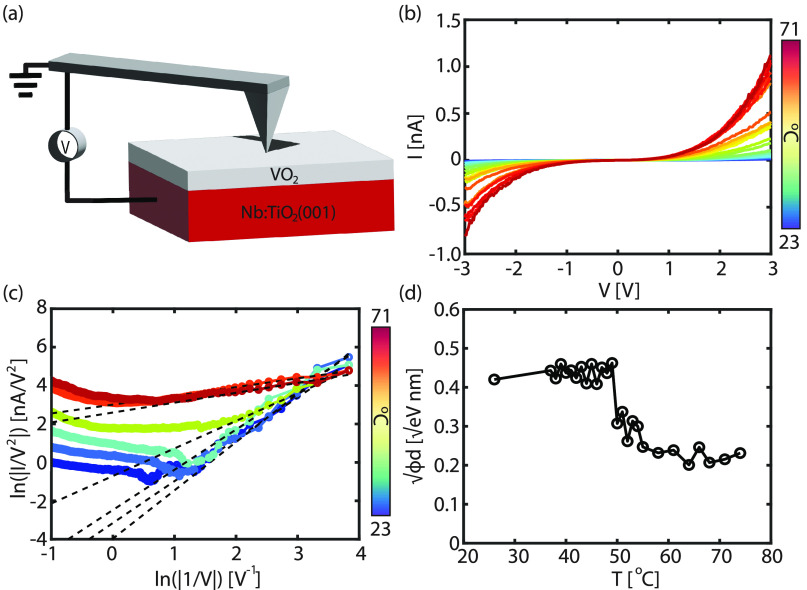
(a) Schematic representation of the experimental setup. The voltage is applied between the tip and the Nb:TiO_2_ substrate. (b) The median *I*(*V*) curves recorded at different temperatures. (c) The same data as in (b) plotted on a ln(*I*/*V*^2^) versus ln(|1/*V*|) scale (only the negative polarity is shown), which is related to the direct tunneling (DT) model. The black dashed lines are fits using eq 1. (d) The obtained barrier height parameters () from panel (c) versus the temperature (*T*). Around 50 °C, a clear transition is observed, which is related to the metal–insulator transition in VO_2_.

Nine to 15 nm thick VO_2_ thin films were grown on 0.5 wt % Nb-doped TiO_2_ (001) substrates (Nb:TiO_2_) by pulsed laser deposition (see the Experimental Section and the Supporting Information for sample preparation and characterization details). This orientation is chosen such that the crystallographic axis of VO_2_ along which the dimerization takes place in the low-temperature phase (*c*-axis in the rutile high-temperature phase) points in the out-of-plane direction. The electronic properties are determined by placing a highly boron-doped, single-crystalline diamond (BDD) AFM tip in direct contact with the VO_2_ film. The amount of load on the sample is controlled by the force feedback, while the current flows between the tip and the Nb:TiO_2_ bottom electrode as a function of the applied voltage (*V*) (see schematic in Figure 1a). The tip acts as the top electrode as well as a nanoscale mechanical indenter. Although the applied pressures are going as high as 8 GPa, the mechanical load is kept below the plastic deformation threshold of the material to avoid permanent deformation or damage to the VO_2_ film (see Supporting Information).

First, the contact properties of the BDD/VO_2_/Nb:TiO_2_ junction are measured as a function of temperature (*T*). The applied pressure during the experiment is kept constant at approximately 2.7 GPa. Local transport measurements reveal a gradual change of the obtained current (*I*) versus voltage (*V*) curves with increasing *T* (see Figure 1b). Because of the semiconducting nature of VO_2_ at room temperature, a Schottky barrier (ϕ_B_) is formed between the conducting tip and the VO_2_ film. In general, the Schottky barrier is dependent on the metal work function (ϕ_M_) and the electron affinity of the semiconductor (χ).^39,40^ However, phenomena like Fermi level pinning can cause a deviation from the standard Schottky–Mott rule.^39,41^ In our experimental structure, a nanoscale (top) electrode is used (AFM tip), and a second macroscopic (bottom) electrode is required (VO_2_/Nb:TiO_2_ interface) to close the electrical circuit. Both electrodes are typically described as two Schottky diodes reversibly connected in series.^42^ Because the tip/VO_2_ contact is much smaller in size compared to the macroscopic contact (9 orders of magnitude), the current blockage by the macroscopic contact is negligible and the nanoscopic contact dominates the current flow. Therefore, our circuit can effectively be described as a single metal–semiconductor junction.^40^

To explain the evolution of the *I*(*V*) curves as a function of temperature, the Schottky barrier height is extracted. There are three carrier injection mechanisms that are dominant in the current flow across a Schottky barrier.^43,44^ First, there is thermionic emission, second, there is direct tunneling (DT), and third, there is Fowler–Nordheim (F–N) tunneling. These mechanisms are influenced by the applied bias configuration, doping level, temperature, and the exact interface composition. In most cases, the carrier injection mechanism across a metal–semiconductor interface is thermionic emission. From the thermionic emission model, both the Schottky barrier height ϕ_B_ and the ideality factor η can be extracted (see Supporting Information for an elaborate explanation and analysis). Both quantities are dependent on the temperature (as shown in Figure S10 in the Supporting Information). For instance, ϕ_B_ increases with increasing temperature while η decreases. The ideality factor is typically used to assess the deviation of the current transport from ideal thermionic emission, where η < 2 implies pure thermionic emission. These trends have also been observed for other material systems, such as for metal electrodes on silicon.^45^ At lower temperatures, the charge carriers have insufficient energy to surmount the Schottky barrier. Therefore, the current transport is dominated by other carrier injection mechanisms, resulting in higher ideality factors. With increasing temperature, more charge carriers gain sufficient energy to overcome the Schottky barrier, resulting in a lowering of the ideality factor. Around 50 °C, a clear change in the slope of the ideality factor is observed. This point coincides with the metal–insulator transition temperature (*T*_MIT_) obtained from the macroscopic transport measurement (see Figure S15 in the Supporting Information).

The large ideality factors obtained from Figure 1b (η ≫ 2) (details are shown in Figures S10–S12 in the Supporting Information) imply that other charge injection mechanisms beyond thermionic emission play an important role.

The DT current depends linearly on the bias according to^46,47^
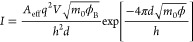
1where *A*_eff_ is the effective contact area of the tip/VO_2_ junction, *m*_0_ the free electron mass, *q* the electronic charge, *h* the Planck constant, *d* the barrier width, and ϕ the height of the tunnel barrier.

F–N tunneling is described by^46,47^
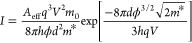
2where m* is the effective mass of an electron (≈ 3.4 *m*_0_(48)).

Equations 1 and 2 imply that DT and F–N differ in terms of *I*(*V*) dependency. Both mechanisms can easily be distinguished when plotting ln(*I*/*V*^2^) as a function of 1/*V*, a so-called F–N plot. For F–N, the curve should decrease linearly, whereas for DT, it should increase logarithmically.^44,46,47^ DT shows a linear behavior when plotted on a ln(*I*/*V*^2^) versus ln(|1/*V*|) scale. For DT and F–N, it is possible to determine the barrier parameters ϕ^3/2^*d* and √*ϕd*, respectively. Both parameters are expressed in terms of the barrier width *d* because the exact width of the barrier is unknown. From the F–N plot (see Figure S10 in the Supporting Information) it is clear that DT is the dominant charge injection mechanism and that F–N plays a minor role in the charge transport here. The inflection point,^44,46,47^ which is defined as the point where the two regimes are separated, is located at relatively high voltages (*V* > 2.5 V). Within this study, the F–N regime cannot be reached, as voltages larger than 3 V permanently degrade the material.^35^

In Figure 1c, the data from Figure 1b are plotted on a ln(*I*/*V*^2^) versus ln(|1/*V*|) scale, also known as a DT plot. A clear linear regime is observed, which can be fitted with eq 1 to extract the DT barrier parameters. The extracted barrier parameter √*ϕ d* is plotted as a function of temperature in Figure 1d. A clear transition is observed around 50 °C. Before the transition, √*ϕd* is approximately constant, which is expected, as tunneling is a temperature-independent process.^49,50^ The average value 0.40 eV^1/2^ nm agrees well with other measurements performed on semiconductors.^44^ Note here that the total current through the junction is still temperature-dependent, as the contribution from thermionic emission is increasing with temperature (decreasing η, see Figure S10 in the Supporting Information).

Surprisingly, after the metal–insulator transition, the barrier height parameter (√*ϕd*) still has a finite value (√*ϕd* ≈ 0.2 eV^1/2^). For a metal–metal contact, no barrier should be present, and the corresponding *I*(*V*) curve should be metallic, i.e., linear. The red curves in Figure 1b clearly deviate from linear behavior. As mentioned in the beginning, for most thin films and bulk surfaces, the dominant current injection mechanism is thermionic emission,^40,41,51,52^ which is contrary to DT being the dominant injection mechanism in our VO_2_ thin films. In general, DT and F–N are the dominant current injection mechanisms in nanosheets or two-dimensional materials.^43,44,47,53,54^

Recent X-ray photoemission (XPS) studies revealed that the surface layer of VO_2_ contains V ions in a 5+ oxidation state, which is larger than the 4+ oxidation state that is expected for the bulk of stoichiometric VO_2_.^55^ Wahila et al. furthermore report the absence of a structural phase transition at the surface of VO_2_ (001) based on low-energy electron diffraction measurements.^56^ For simplicity, we use the common rutile crystallographic basis for both the substrate and the thin film, as it applies to the latter above the phase transition temperature. Wagner et al.^57^ have studied the surface of VO_2_ (110) in great detail and report an oxygen-rich reconstruction of the VO_2_ surface reminiscent of V_2_O_5_, a band insulator that does not possess a metal–insulator transition. As the rutile (110) surface is actually the lowest energy surface of VO_2_, we suspect that similar oxidation also happens at the other higher energy, and hence less stable, surfaces.

To test this hypothesis, we performed XPS and laboratory-source hard X-ray photoemission spectroscopy (HAXPES) measurements with different takeoff angles on our VO_2_ sample. In phase pure VO_2_, we expect an oxidation state of 4+ for all vanadium ions. However, previous reports have shown that the surface of VO_2_ is more accurately described by an oxidation state of 5+ due to spontaneously formed, oxygen-rich surface reconstructions.^55,57−59^

**Figure 2:**
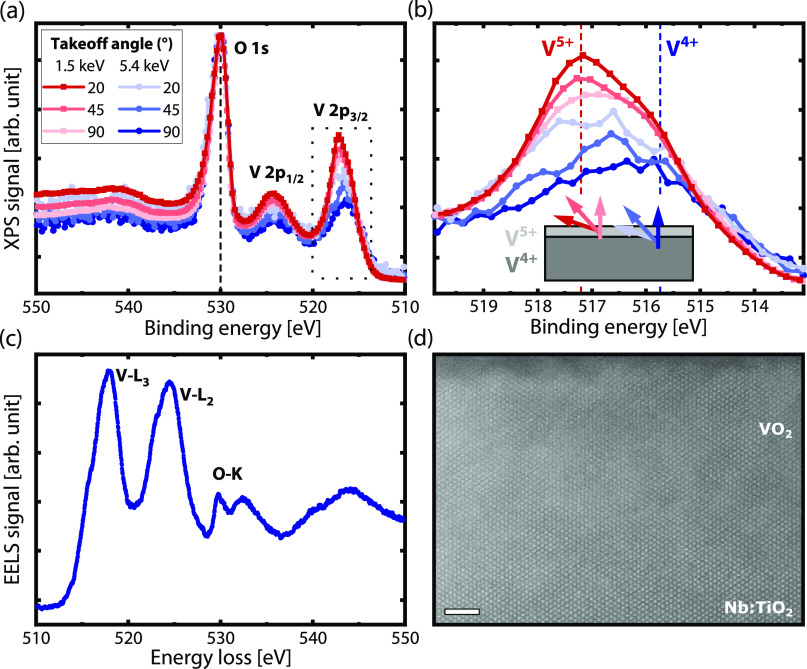
X-ray photoemission measurements using two different photon energies and three different takeoff angles. The inset in panel (b), which is a zoom of the dotted area in panel (a), shows how the takeoff angle is defined with respect to the sample surface. Indicated with red and blue dashed lines are the expected peak positions for V^5+^ (517.2 eV) and V^4+^ (515.74 eV) according to Silversmit et al.^60^ At the surface, the V valence is higher (5+) than in the bulk (4+). (c) Electron energy loss spectroscopy (EELS) signal of the interior of the VO_2_ layer. (d) Atomic resolution scanning transmission electron microscopy image of the sample cross-section in the [100] zone. The length of the scale bar is 2 nm.

We summarize our XPS/HAXPES results in Figure 2. At the surface, we find a 5+ oxidation state for vanadium indeed, fully in agreement with the earlier reports mentioned. By increasing the probing depth of our experiment, which we achieve by increasing the incident photon energy (switching from an Al anode to Cr) and increasing the photoelectron takeoff angle, the V 2p peaks shift toward lower binding energies (see Figure 2a). Such a shift is characteristic of a reduced oxidation state. The expected location of the V 2p_3/2_ peak for V^5+^ is at a binding energy of 517.2 eV, whereas for V^4+^, it is reduced by 1.46 eV to 515.74 eV.^60^ As we indicated by the dashed reference lines of Figure 2a and b, the magnitude and the direction of the peak shift are consistent with the proposed model. In the model, it is assumed that the interior of the VO_2_ film has the expected vanadium oxidation state of 4+, and the surface has a higher 5+ oxidation state. We pictorially summarize this observation in the cartoon inset in the bottom panel of Figure 2b.

Unlike tetravalent vanadium oxide (VO_2_), pentavalent vanadium oxide (V_2_O_5_) is a band insulator that does not possess a metal–insulator transition. V_2_O_5_ has a band gap of 2.2 to 2.4 eV due to the empty 3d orbital.^61^ We conclude that it is this pentavalent, insulating surface layer that leads to the tunneling-dominated *I*(*V*) characteristics observed by C-AFM. Using this information as input, we can estimate the magnitude of the DT barrier parameter. The value for the energy barrier ϕ is typically assumed to be half of the band gap of the insulating layer, here ∼1.2 eV, and we can reasonably assume the thickness of the insulating surface layer to be on the order of one unit cell of VO_2_ (001), i.e., ∼0.28 nm. The product of the square root of the energy barrier multiplied by the tunneling distance is then ∼0.3 eV^1/2^ nm. This is well in accordance with the experimentally obtained DT barrier parameters shown in Figure 1d.

The insulating V^5+^ surface layer remains present after the semiconductor-to-metal transition of the interior of the VO_2_ film and dominates the charge injection mechanism before and after the semiconductor-to-metal transition. However, the structure and electronic properties of the single unit cell layer heavily depend on the structure of the VO_2_ thin film. First, in the semiconducting (monoclinic) phase, a tunnel barrier is present in series with a Schottky barrier. For the metallic (rutile) phase, the Schottky barrier vanishes due to the high free carrier density, and only the tunnel barrier is present, significantly lowering the barrier width *d*. Second, most likely, the small shifts of the atomic positions in the VO_2_ lattice also lead to small changes in the structure and electronic properties of the pentavalent vanadium oxide surface layer. Analogous effects are also observed for other thin layers.^62^ These two effects both influence the barrier height parameter of the tunneling process, and therefore a transition is observed in the current–voltage spectroscopy.

Our photoemission study was performed on samples that were exposed to the ambient air just like the samples studied by C-AFM, i.e., *ex situ*. We would like to note that even in samples that are transferred in ultrahigh vacuum from the PLD growth to the XPS analysis chamber without exposure to ambient air (*in situ*), we still observe a significant V^5+^ contribution to the expected V^4+^ signal. Details on the XPS/HAXPES experiments are provided in the Supporting Information.

To further investigate the interface between the substrate and the thin film and to confirm the oxidation state of vanadium, we performed scanning transmission electron microscopy (STEM) and electron energy loss spectroscopy (EELS). A cross-sectional lamella was prepared by focused ion beam (FIB) milling. In addition to the position of the V L_2,3_ edge, another indicator for the oxidation state and the monoclinic phase of VO_2_ at room temperature, i.e., below the phase transition, is the fine structure of the O K-edge.^63^ In VO_2_, the latter is composed of three features, namely, the overlapping π* and d_∥_^*^ states that form the asymmetric peak around 530 eV and the σ* states that follow around 532.5 eV energy loss. Away from the top surface, which may have been damaged by the FIB processing, we find a clear V^4+^ character for the interior of the VO_2_ thin film; see Figure 2c. This is in full agreement with earlier reports by Tashman et al.^64^ and Lu et al.^65^ In accordance with the XRD analysis, no crystalline phases other than the expected, commensurately strained VO_2_ are observed. This is illustrated by the atomic resolution high-angle annular dark-field (HAADF) image displayed in Figure 2d, which evidences the epitaxial registry of the thin film and the substrate. Further details on the STEM-EELS analysis and an EELS line profile are provided in the Supporting Information

A brief recap, the metal–insulator transition is observed in VO_2_ films using a conductive AFM tip while varying the temperature. Even when the interior of the film becomes metallic, the interface layer acts as a small barrier between the conductive tip and the VO_2_ thin film, resulting in direct tunneling as the dominant transport mechanism.

Besides monitoring the change of the contact mechanism with a varying temperature at a constant pressure, a similar experiment can be conducted by varying the applied pressure at a constant temperature. In this case, the load applied by the AFM tip on the VO_2_ surface is varied, and simultaneously the *I*(*V*) characteristics are measured. This enables the opportunity to measure the current injection mechanism under different applied pressures, including the metal–insulator transition. Furthermore, rastering allows for recording spatial maps and subsequent averaging over local differences in topography.

**Figure 3:**
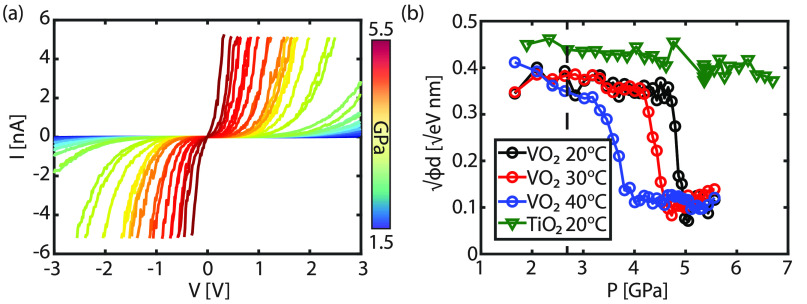
(a) *I*(*V*) curves recorded with a doped diamond tip on VO_2_ under different applied pressures at 50 °C. (b) The obtained barrier height parameter () using eq 1 versus the applied pressure (*P*) measured at different temperatures (black = 20 °C, red = 30 °C, blue = 40 °C). A clear transition is observed for all three temperatures. The green line is a reference measurement of TiO_2_, which is a material with no metal–insulator transition. The dashed black line is the applied pressure for the varying temperature measurement in Figure 1d.

In Figure 3a, the evolution of the *I*(*V*) characteristics for a BDD/VO_2_/Nb:TiO_2_ junction at 50 °C is shown for pressures varying between 1.5 and 5.5 GPa. Similar to the varying temperature experiment, an increase in current is observed for increasing pressures. As a reference measurement, the experiment is repeated on a conventional semiconductor (TiO_2_, see Supporting Information for sample preparation and characterization details). Similar to VO_2_, the obtained ideality factor is large (η > 4), indicating that other charge injection mechanisms are more dominant compared to the thermionic emission model. Using the F–N plot, we find that the inflection point is absent, indicating that direct tunneling is again the dominant charge injection mechanism at the used bias voltages. The extracted barrier height parameter is plotted as a function of pressure for TiO_2_ (green triangles in Figure 3b). A monotonic decrease of the barrier height parameter is observed. As the barrier height parameter is a function of the product of the barrier height (ϕ) and the barrier thickness (*d*), and both parameters are affected by the applied pressure, the exact influence of either parameter cannot be individually analyzed. For instance, the band gap of the material may change with applied pressure,^66,67^ affecting ϕ, while *d* is also reduced with increasing pressure. However, most likely, the reduction of *d* is the main mechanism behind the reduction of  in TiO_2_, as the tunneling current is exponentially dependent on *d*. In addition, no abrupt change in the barrier height parameter is observed as a function of pressure in the case of TiO_2_.

Besides TiO_2_, the barrier height parameter (√*ϕd*) is also obtained for VO_2_ as a function of pressure for different temperatures; see Figure 3b. In contrast to TiO_2_, a clear transition is observed in √*ϕd*, which is dependent on the temperature. At low applied pressures, √*ϕd* is decreasing slightly with pressure. Most likely, this reduction is caused by the small decrease in the barrier width *d*, similar to TiO_2_. At higher pressures (*P* > 3 GPa), however, an abrupt transition is observed. The observed transition as a function of pressure is very similar to the transition observed within the varying temperature experiment (see Figure 1), except for the absolute √*ϕd* values of the barrier parameter after the MIT. The barrier parameters after the MIT are 0.2 and 0.1 eV^1/2^ nm for a varying temperature and pressure, respectively. The discrepancy in the barrier parameter reveals that both temperature and pressure have a different influence on the contact mechanism. As the temperature is increased, the structure and thereby also the electronic properties of the surface layer change. Under applied pressure, however, additional compression is introduced into the interfacial layer, reducing the barrier width even more. In other words, an increase in the pressure not only induces the MIT but also reduces the effective barrier width.

**Figure 4:**
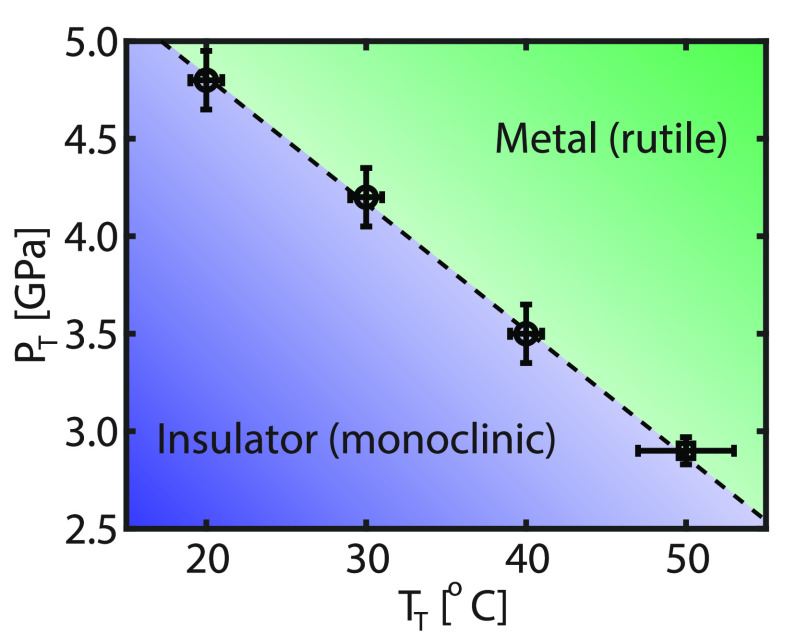
Transition pressure (*P*_T_) versus the transition temperature (*T*_T_). The measurement points are extracted from the varying temperature (Figure 1, ○) and varying pressure (Figure 3, □) experiment.

When the temperature of the VO_2_ is increased, the applied pressure required to induce the metal–insulator transition decreases (see Figure 3b). From the varying temperature (Figure 1) and pressure (Figure 3) experiment, the transition pressure (*P*_T_) and the transition temperature (*T*_T_) are extracted. In Figure 4 the transition pressure as a function of the transition temperature is shown.

Along the coexistence line (dashed line in Figure 4), both phases exist at a constant pressure and temperature. Using the Gibbs–Duhem equation, one can derive the Clapeyron relation
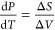
3with Δ*S* the entropy and Δ*V* the volume changes across the solid–solid phase transition. The entropy per volume ratio can be determined from the slope of the coexistence line. From the slope (linear regression), a value of 60 MPa °C^–1^ is found, which is in excellent agreement with previously reported values for macroscopic measurements on the phase transition of VO_2_.^27,68^

What makes pressure-controlled C-AFM exciting for the study of quantum materials is the enormous range of pressures that can conveniently and routinely be accessed under the sharp probe apex and the resulting giant changes in the local structural and electronic properties of the material this can unleash. Assuming a Young’s modulus of 128 GPa,^69^ we linearly convert stress into strain using Hooke’s law in its one-dimensional scalar form. The transition pressures of 3 to 5 GPa displayed in Figure 4, which are needed to drive the MIT of VO_2_ by nanoindentation, then correspond to vast uniaxial compressive strains along the rutile *c*-axis of approximately 2% to 4%. Although the exact pressure distribution in AFM-based nanoindentation is more complicated than one-dimensional compression, we confirmed with finite element modeling in the Supporting Information that the uniaxial out-of-plane compression term dominates over lateral effects and indeed extends throughout the thickness of our films.

Strains of several percent are roughly 1 order of magnitude more than what bulk oxide specimens can usually withstand before cracking and thus greatly expand the iconic phase diagram of Park et al.^27^ In addition, when down-scaling electronics toward the nanoscale, the contacts become increasingly more important. Using an AFM tip as an electrode, the dominant current injection mechanisms can be identified for different materials and even for phase transitions within the material at the device-relevant length scales.

## 3 Conclusions

To conclude, we have demonstrated that by controlling the applied pressure with an AFM tip, we can noninvasively manipulate and control the electronic metal–insulator transition of VO_2_. Furthermore, we have shown that the metal–semiconductor junction plays an important role in the conduction mechanism of the nanometer-sized contact. Such mechanical tunability allows the experimental determination of the contact properties as well as the coupled structural and electronic metal–insulator transition. Specifically, the MIT in VO_2_ is observed in the barrier height parameter with varying temperature and pressure, respectively, in a hitherto inaccessible regime of the phase space. Using a pressure of approximately 5 GPa, we can reversibly and deterministically trigger the MIT at room temperature and mechanically switch the material locally, i.e., on the nanoscale under the probe tip, from an insulating to a conductive state. At all times, an intrinsically insulating surface layer is inevitably present between the metal tip and the bulk-like interior of the VO_2_ thin film. Therefore, the dominating current injection mechanism is direct tunneling, which remains unaltered during the transition. The barrier height parameter changes significantly and abruptly during the metal–insulator transition. With varying temperature, only the band structure of the VO_2_ is changing, while with varying pressure, the thickness of the interfacial layer is affected in addition to the band structure. The combination of heteroepitaxial in-plane confinement due to fully coherent crystal growth of a thin layer onto a slightly mismatched substrate with local, uniaxial-like out-of-plane strain due to nanoindentation with a scanning probe tip allowed us to enter a hitherto inaccessible and unexplored region in the pressure–temperature phase diagram of VO_2_ and serves as a versatile technique to study quantum materials under symmetry-breaking stresses that can easily be extended toward very high electric fields and large stresses, as well as spatial mapping of local differences in micro- and nanostructures. Our findings show that C-AFM is capable of measuring the contact properties of nanoscale junctions during phase transitions and to extract the dominant charge injection mechanisms as well as the barrier height parameters, which is valuable information for the development of future nanoscale metal-oxide devices.

## 4 Experimental Section

### 4.1 Sample Preparation and Structural Characterization

The thin films are deposited via reflection high-energy electron diffraction (RHEED)-assisted pulsed laser deposition (PLD) onto 0.5 wt % Nb-doped TiO_2_ (001) substrates purchased from CrysTec GmbH. X-ray diffraction reciprocal space mapping is used to ascertain the coherent epitaxial relation between film and substrate and to extract the lattice parameters. Details of the sample preparation as well as the XPS/HAXPES and STEM-EELS analysis are reported in the Supporting Information.

### 4.2 AFM Measurements

All the C-AFM measurement are performed using a Bruker Dimension Icon AFM in a N_2_ environment by continuously purging with N_2_ gas, to avoid discrepancies in the data and water-induced reactions.^35^ The relative humidity (RH) was measured using a humidity sensor (SENSIRION EK-H4 SHTXX, Humidity Sensors, Eval Kit, SENSIRION, Switzerland), with an accuracy of 1.8% between 10% and 90% RH. With the DCUBE mode, both current–voltage (*I*(*V*)) and force–distance (*F*(*D*)) curves are obtained and form a hyperspectral data set. Within this mode, the force is constantly regulated and recorded. The voltage is applied to the sample while the tip is grounded. The measurements are performed with highly boron-doped diamond tips (AD-2.8-AS and FM-LC; Adama Innovations Ltd., resistivity: 0.003 to 0.005 Ω cm). The sample is heated using a platinum resistive-type heater in a ceramic body and a tungsten cap controlled by a thermal applications controller. The samples are glued with silver paint on a metallic plate that is magnetically mounted onto the heater. A thermocouple is also mounted on the metallic plate to determine the temperature of the sample. In order to determine the pressure with which the AFM tip is pressing on the sample surface, the contact area is needed. The contact area of the tip is acquired using the DMT theory.^70^ For more detailed information see the Supporting Information The error analysis for the measurements in Figures 1d and 2b can also be found in the Supporting Information.

## Data Availability

The data sets generated during the current study are available from the corresponding author on reasonable request.

## Supporting Information Available

Additional experimental details, materials, methods, finite element model, and error analysis (PDF)
