# Supplementary material for: High-Strain-Induced Local Modification of the Electronic Properties of VO2 Thin Films
Source: ACS Appl Electron Mater. 2022 Nov 18;4(12):6020–8. doi: 10.1021/acsaelm.2c01176 (PMC9798830; doi:10.1021/acsaelm.2c01176)
Supplement: Supplementary file 1 — el2c01176_si_001.pdf [file el2c01176_si_001.pdf]

# Supporting Information: High-strain-induced local modification of the electronic properties of $VO_2$ thin films

Yorick A. Birkhölzer,[*,†,‡] Kai Sotthewes,[*,†,‡] Nicolas Gauquelin,[¶] Lars Riekehr,[¶] Daen Jannis,[¶] Emma van der Minne,[†] Yibin Bu,[†] Johan Verbeeck,[¶] Harold J.W. Zandvliet,[†] Gertjan Koster,[†] and Guus Rijnders[†]

†*MESA+ Institute of Nanotechnology, University of Twente, P.O. Box 217, 7500AE Enschede, The Netherlands*

‡*Contributed equally*

¶*Electron Microscopy for Materials Science (EMAT), University of Antwerp, Groenenborgerlaan 171, 2020 Antwerp, Belgium*

E-mail: y.a.birkholzer@utwente.nl; k.sotthewes@utwente.nl

# Contents


**1 Sample preparation** **S-3**

**2 Structural characterization** **S-5**

**3 Tip and sample parameters** **S-7**

**4 Finite element model** **S-9**

**5 Acquisition of the $I(V)$ curves** **S-12**

**6 Stability of the $VO_2$ layer** **S-13**

**7 Contact area** **S-13**

**8 Charge injection mechanisms** **S-16**

**9 Error analysis** **S-18**

**10 Thermionic emission data** **S-18**

**11 X-ray photoemission spectroscopy** **S-23**

**12 Scanning transmission electron microscopy** **S-25**

**13 Macroscopic transport measurements** **S-27**

References **S-29**

# 1 Sample preparation

Single crystalline substrates of 0.5 wt.% Nb-doped rutile $TiO_2$ (001) (5 x 10 x 0.5 mm, miscut $< 0.2°$) were purchased from CrysTec GmbH. Prior to thin film growth, the substrates were cleaned in an ultrasonic bath for 5 minutes first with acetone and second with isopropanol. Subsequently, they were annealed for 120 minutes at 800 °C in a tube furnace under an oxygen flow of 150 L h$^{-1}$. The resulting surface had a root mean square (rms) roughness of 0.13 nm as determined by ambient tapping mode AFM (see Fig. S1) before loading the sample into the pulsed laser deposition (PLD) tool. Preliminary attempts to form an atomically flat surface with vicinal steps and terraces by annealing at 850 °C (after 1 minute of etching in buffered HF) or 950 °C for 90 minutes lead to complete destruction of the smoothly polished surface and excessive faceting.

The PLD used has a base pressure better than $2 \times 10^{-7}$ mbar. Using a rectangular mask (3 x 8 mm$^2$), a mirror and a lens, 248 nm wavelength UV light from a KrF excimer laser (COHERENT COMPex Pro 205 F, pulse duration 25 ns) was sharply imaged onto a raster scanning polycrystalline $V_2O_5$ target (20 mm diameter) with a repetition rate of 4 Hz. The spot size on the target was 1.75 mm$^2$. A manual attenuator in the beam path was used to adjust the laser fluence to 1.3 J cm$^{-2}$. Before loading the substrate into the chamber, the target (ground with 1000 grit sandpaper) was pre-ablated with 2000 laser pulses. The films were grown at a temperature of 400 °C (measured by a thermocouple inside the resistive heater element) and under a dynamic background pressure of 0.01 mbar oxygen gas. This rather low temperature was chosen with the intention to create a sharp interface and limit titanium diffusion into the film. After deposition, the sample was cooled down to room temperature at a rate of 10 °C min$^{-1}$ under the deposition pressure. No additional annealing step was performed.

The PLD is equipped with *in situ* high-pressure reflection high-energy electron diffraction (RHEED). In Fig. S2, we show the diffractograms recorded at 30 kV in 0.01 mbar $O_2$ along the [100] direction of the (001) rutile substrate before and during $VO_2$ film growth. The sharp

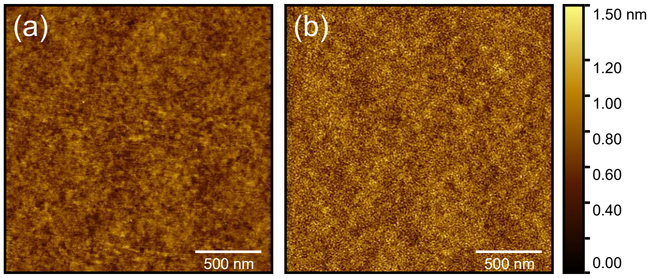


Figure S1: AFM Topography images (a) before (rms roughness 0.13 nm) and (b) after (rms roughness 0.18 nm) $VO_2$ film growth.

spots and the Kikuchi bands attest to a high surface quality. We monitored the intensity of the specular spot during deposition (not shown) but did not observe any intensity oscillations that would be expected from layer-by-layer growth. The observed diffractogram after film growth is a typical transmission pattern characteristic of an island growth mode, consistent with the absence of intensity oscillations in the specular spot. It is likely that the unfavorably high surface energy of the (001) plane is the underlying cause for promoting the island growth mode and associated surface roughening.[1,2]

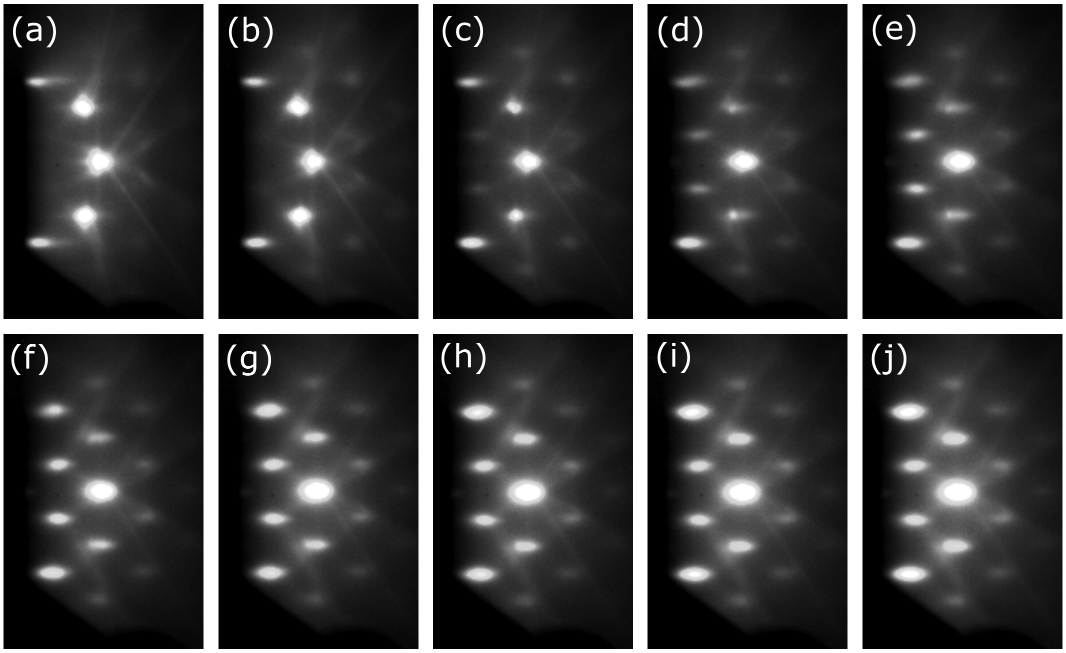


Figure S2: RHEED patterns taken before (a) and during deposition at approx. (b) 2, (c) 3, (d) 4, (e) 5, (f) 7, (g) 20, (h) 30, (i) 40, (j) 53 (end of the deposition) unit cells of $VO_2$ film growth.

## 2 Structural characterization

High-resolution X-ray diffraction (XRD) was performed using a Bruker D8 Discover equipped with a high brilliance microfocus rotating anode generator (TXS, 2.5 kW), an asymmetric channel-cut Ge (022) two-bounce monochromator, a 1 mm pinhole collimator, and a large Eiger2 R 500k area detector with high dynamic range.

The $VO_2$ film thickness was determined by fitting the Laue fringes observed in a $2\theta - \omega$ scan (see Fig. S3 (a)) via a dynamical X-ray diffraction simulation using the program *gid_sl* on Sergey Stepanov's X-ray server (`https://x-server.gmca.aps.anl.gov`).[3] In the calculation, we assume an interface roughness of 0.2 nm consistent with the AFM measurements shown in Fig. S1. It is those sharp interfaces combined with the high crystallinity that enable the observation of multiple Laue fringes. For a deposition time of 1000 seconds with 4000 laser pulses at 4 Hz a film thickness of $15.0 \pm 0.1$ nm is extracted, resulting in a growth rate of $0.015\,\mathrm{nm\,s^{-1}}$ which corresponds to $75 \pm 1$ pulses per unit cell.

Three-dimensional reciprocal space maps (RSM) were constructed from sets of rocking curves measured in coplanar geometry. Projections summed over one of the in-plane momentum axes are shown in Fig S3 (b - c). From these RSMs the following rutile-like lattice parameters for the $VO_2$ film are extracted via 2D Voigt function fitting of the thin film peak: $a = b = 4.5937 \pm 0.0010\,\text{Å}$, $c = 2.8275 \pm 0.0050\,\text{Å}$.

As films with a thickness of 15 nm or more are prone to cracking over time to relax the strain, the remainder of this study, in particular the AFM, XPS/HAXPES, and STEM-EELS experiments, was performed on thinner films with a thickness of only 10 nm.

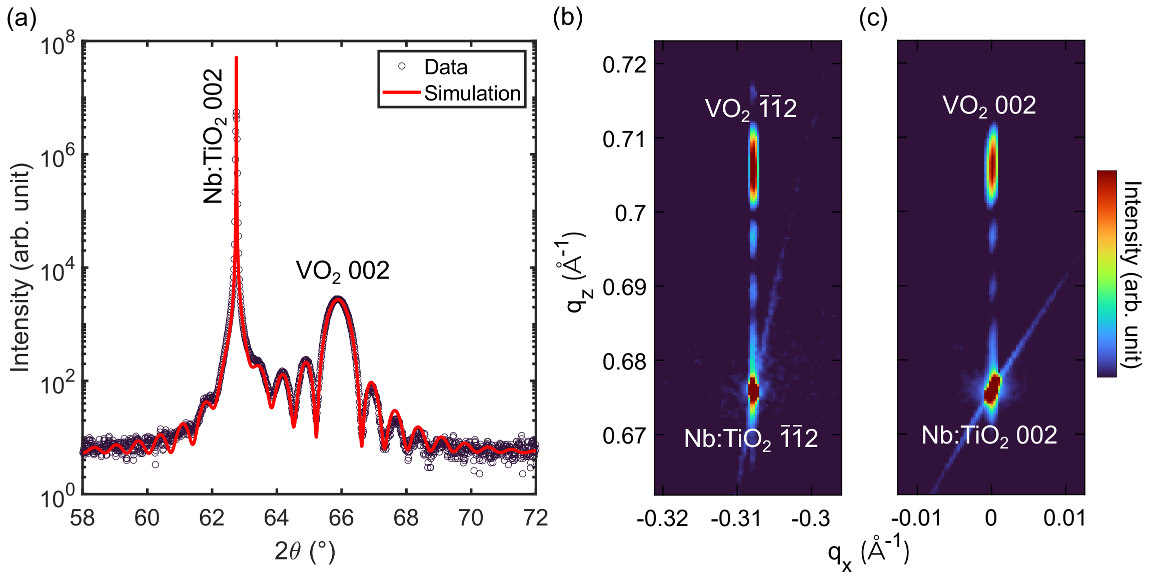


Figure S3: X-Ray diffraction. (a) $2\theta-\omega$ scan with a superimposed fit. (b) Asymmetrical and (c) symmetrical reciprocal space maps of the $\bar{1}\bar{1}2$ and 002 reflections confirming the single-oriented growth and coherently strained epitaxy as evidenced by the identical in-plane ($q_x$) momentum transfers for substrate and thin film.

# 3 Tip and sample parameters

In Table 1 and 2, the material properties of the boron-doped diamond tips (purchased from Adama Innovations Ltd., Ireland) and the sample are summarized. The work function of the diamond tip in Table 1 is characterized using Kelvin Probe Microscopy. The radius is confirmed by SEM measurements to be within the range specified by the supplier, namely $r = 10 \pm 5\,\mathrm{nm}$ (see Fig. S4). The spring constant is determined by using the thermal vibration method.[4] First, the deflection sensitivity is determined on a sapphire sample, and subsequently, the spring constant is measured from the thermal tuning.

By measuring the I(V) characteristics of a Au film (not shown) and confirming that it is ohmic as expected, we verified that the tunneling behavior shown in the main article is indeed a property of the $VO_2$ - tip junction, and not a property inherent to the boron-doped diamond tip alone.

Table 1: Various cantilever properties including the Young's modulus ($Y$), Poisson ratio ($\nu$), spring constant ($k$), work function ($\phi_{\mathrm{M}}$) and tip radius ($r$). The spring constant is determined using the thermal vibration method.[4]

| | $Y$ [GPa] | $\nu$ | $k$ [N/m] | $f_{\mathrm{r}}$ [kHz] | $\phi_{\mathrm{M}}$ [eV] | $r$ [nm] | $\chi$ [eV] |
|---|---|---|---|---|---|---|---|
| AD-2.8-AS | 1000[5] | 0.07[5] | 8 | 65 | 5.1 | 15 | 0.02[6] |
| FM-LC | 1000[5] | 0.07[5] | 3 | 65 | 5.1 | 30 | 0.02[6] |

Table 2: Various bulk material properties including the Young's modulus ($Y$), Poisson ratio ($\nu$), band gap ($E_{\mathrm{G}}$) and electron affinity ($\chi$).

| | $Y$ [GPa] | $\nu$ | $E_{\mathrm{G}}$ [eV] | $\chi$ [eV] |
|---|---|---|---|---|
| $VO_2$ | 90-150[7–10] | 0.249[11] | 0.6-0.7[12,13] | 2[14] |
| $TiO_2$ | 151[15] | 0.27[15] | 3.1[16] | 3.43[17] |

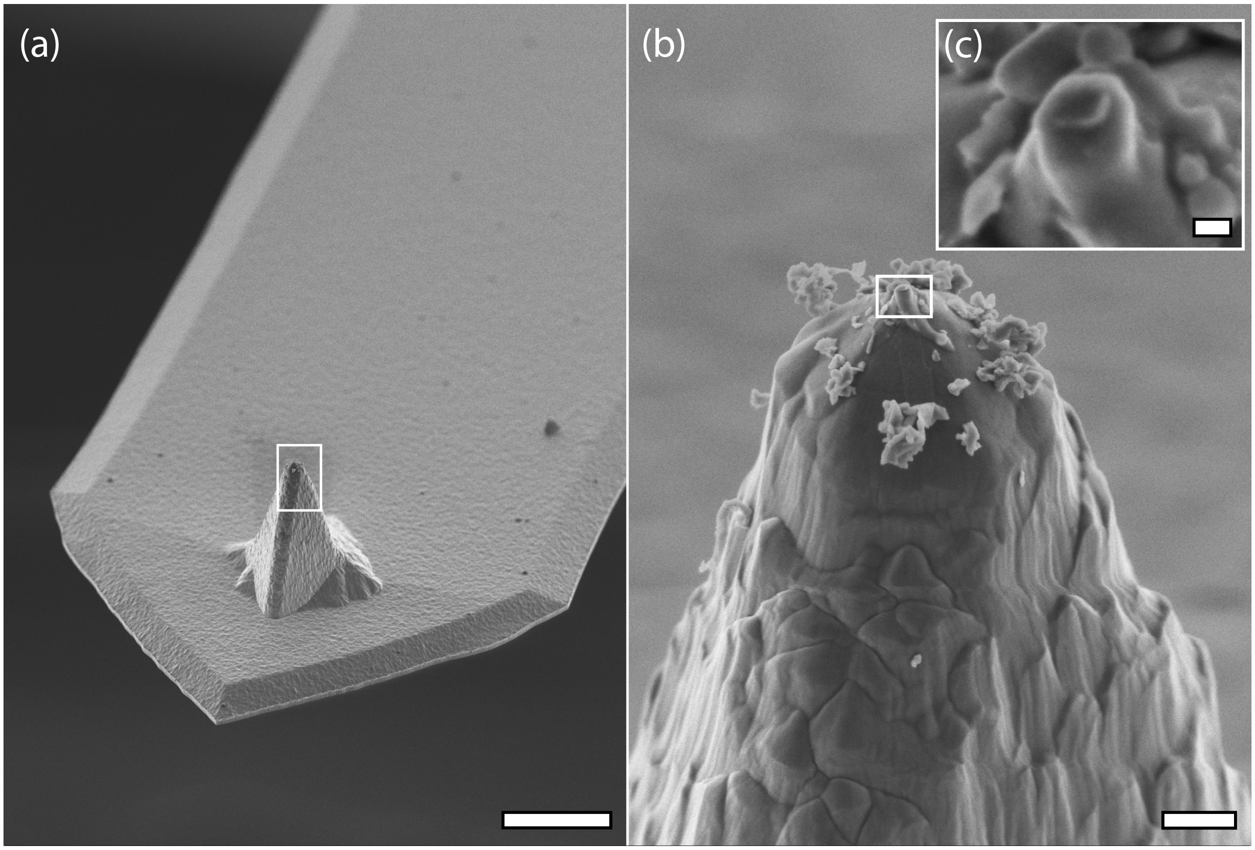


Figure S4: Scanning electron micrographs of the tip used for this study (AD-2.8-AS). Panel (b) is a zoom of the area indicated by the white rectangle in (a). Panel (c) is an even higher magnification of the area indicated by the white rectangle in (b), albeit recorded at a slightly different angle. The scale bars in panels (a), (b), and (c) measure 10 µm, 1 µm, and 50 nm, respectively.

# 4 Finite element model

A finite element model of the nanoindentation process is made in COMSOL Multiphysics 5.6 using the *MEMS Module*. The indentation process is implemented via a parametric deformation study. The apex of the AFM probe is modeled as a half-sphere, which is indented into the stationary $VO_2$ layer. The bottom of the $VO_2$ layer is constrained by fixed boundary conditions, whereas the tip of the indenter undergoes a prescribed parametric displacement downwards into the film. The sides are not constrained in the simulation. 2D axisymmetry along a vertical line is applied to the simplified cross-sectional geometry, see the drawing in Fig. S5 (a). As usual, the mesh is refined near the contact line and finer for the impacted surface than for the indenter. Triangular elements are used for the indenter and rectangular elements for the film. For simplicity, the Nb-doped $TiO_2$ substrate is omitted, but to nevertheless assure a homogeneous stress and strain distribution far away from the indenter, the film is modeled much thicker in the finite element simulation than in the actual experiment described in the main article. The materials parameters used in the simulation are the same as summarized in the tables in the previous section of this supplemental material. The simulation does not include the effects of adhesion as these were beyond the scope of our experimental investigation.

The stress and strain distributions resulting from an indentation of 1 nm are depicted in Fig. S5 (b) - (d). The peak compressive out-of-plane strain $\epsilon_{zz}$ exceeds 16 % locally under the tip, but more importantly for this study, the entirety of a $\sim$ 10 nm thick layer experiences a compressive out-of-plane strain of more than 2 %, which we expect to suffice to induce the phase transition in $VO_2$ at room temperature based on an extrapolation of the published pressure-temperature phase diagram by Park et al.[18]. Not shown here are the in-plane strains and stresses, which are much smaller than the relevant out-of-plane counterparts.

We would like to note that more than half of the strain needed to trigger the phase transition in $VO_2$ at room temperature (2 % compression) is already statically and non-reversibly provided by epitaxy, as the $VO_2$ thin film is commensurately strained onto the

Nb-doped $TiO_2$ (001) substrate. As shown by our XRD measurements, the lattice mismatch leads to a contraction of the rutile c-axis of the $VO_2$ thin film of $\sim 1.2\,\%$. Hence, much less than the 1 nm indentation simulated in Fig. S5 should suffice to demonstrate the pressure-driven semiconductor-metal transition.

Due to the gross simplification of the geometry and some uncertainty in the materials parameters, the simulation should be taken only as a ballpark estimate and not as a quantitatively accurate prediction. We would like to note, however, that the order of magnitude of the calculated stresses and strains are in reasonable agreement with the experimental findings presented in the main article.

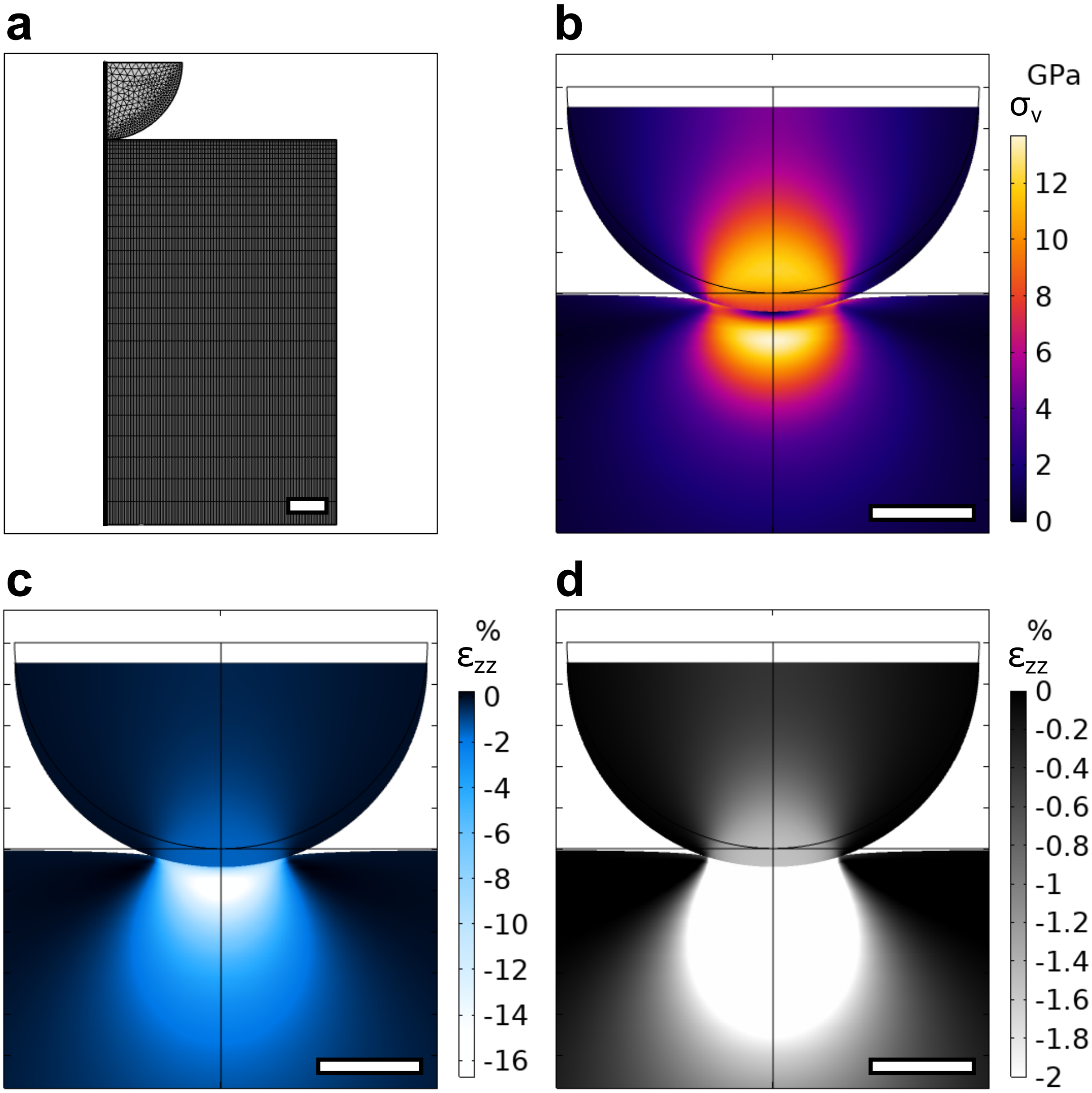


Figure S5: Finite element model. (a) Drawing of the mesh, (b) von Mises stress $\sigma_v$ distribution in the fully indented state, where the tip is displaced by 1 nm, (c) out-of-plane compressive strain $\epsilon_{zz}$, (d) same data as in (c) but on truncated greyscale to emphasize the part of the film which experiences at least 2 % of compressive strain, which is expected to induce the phase transition. The position and shape of the indenter as well as the film prior to the forced displacement are sketched with thin black lines in (b) - (d). The length of the scale bar in all panels is 5 nm.

# 5 Acquisition of the $I(V)$ curves

All the measurements in this study are obtained using the Bruker DCUBE mode. Within this mode, both the force-distance curves ($F(D)$) and current-voltage ($I(V)$) curves are obtained. During the $I(V)$ measurement, the force is actively controlled and monitored. This avoids any change in force during the $I(V)$ measurement. Besides the control during the electrical measurement, also the force during the approach and the retraction is measured. From these force-distance curves, additional information can be extracted, such as the adhesion and the amount of indentation.[19] However, the most important parameter extracted from these measurements is the total work of adhesion which is essential for the determination of the contact area (see section 7).[20]

The $I(V)$ curves shown in the main text are obtained by taking the median over multiple curves. Multiple $I(V)$ curves are taken in a grid-like fashion, in which the scanning area is divided in $12 \times 12$ (or $8 \times 8$) equally spaced positions. At every position, an $I(V)$ measurement is recorded. The average spacing between the spectra is approx. 1 $\mu$m. For each $I(V)$ curve the piezo scanner is paused, and the bias is ramped from -3 V to 3 V to avoid permanent deformation of the sample.[21] From every measurement, the average curve, the median curve, and the raw median curve are extracted from 144 curves in total. The average curve is the mean of all the 144 curves, while the median curve is the median of the same measurements. The raw median curve is determined by determining the median of every single point of the $I(V)$ curve (which is 416 points). The most common curve number is then taken as the raw median curve. As can be seen in Fig. S6, there is no deviation between the median curve and the raw median curve. As the median curve is much smoother, this is the curve used for the analysis in the main text. The average curve deviates from most of the measurements around the saturation point of the I-V converter ($I > 5$ nA in this example) and is therefore not used.

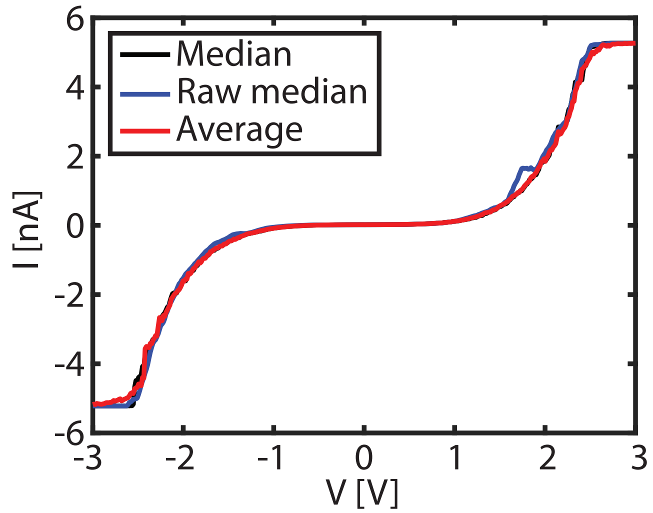


Figure S6: An $I(V)$ curve recorded on $VO_2$ at 50 °C and 4.2 GPa. The median curve is used for the data analysis. The raw median curve is too noisy to be used in the analysis.

.

# 6 Stability of the $VO_2$ layer

After the pressure experiments, part of the surface where the measurements are performed are investigated with an AFM. No permanent deformation or damage is observed to the layer, proving the reversibility of the metal-insulator transition. However, when the surface is scanned with the same force as the $I(V)$ measurements are performed, the $VO_2$ layer is scraped away from the $TiO_2$ substrate. The lateral movement of the tip inflicts damage on the surface and then acts as a scalpel. Therefore, it is crucial to only exert pressure in the vertical direction on the surface and avoid any lateral movement.

# 7 Contact area

In order to determine the applied pressures with which the AFM cantilever presses on the host material, the applied load, and the effective contact area need to be known. The applied load is controlled by the measurement sequence described in section 5. The effective contact area can be derived from contact-mechanic models such as the Hertzian model, which assumes a hemispherical tip shape and neglects adhesive forces.[22]

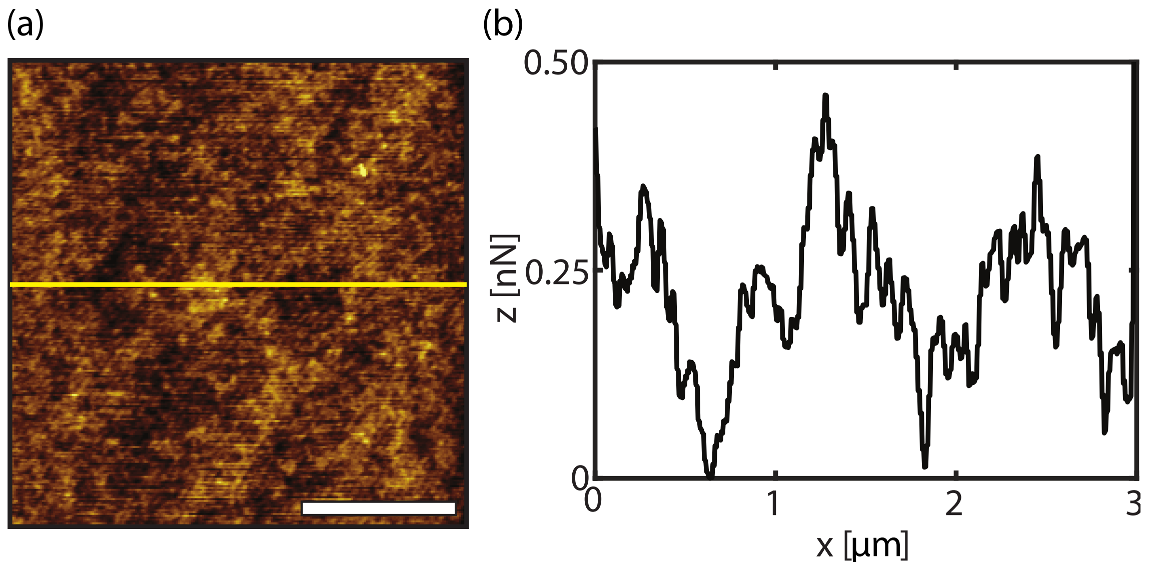


Figure S7: (a) Topographic image (3 × 3 $\mu$m, scale bar 1 $\mu$m) of the $VO_2$ surface after a pressure experiment. No permanent deformation or damage is observed after the experiment. (b) Cross-section height profile of the yellow line in (a).

.

Based on the Hertz equation, the physical contact area radius of the tip on a surface is given by

$$a = \sqrt[3]{RFK} \tag{S1}$$

where $R$ is the radius of the tip, $F$ the applied load, and $K$ is the combined elastic modulus of the tip and sample, given by $K = 4/3((1-\nu_\mathrm{t}^2)/Y_\mathrm{t})^{-1} + ((1-\nu_\mathrm{s}^2)/Y_\mathrm{s}))^{-1}$ where $Y_\mathrm{t}$, $\nu_\mathrm{t}$ $Y_\mathrm{s}$ and $\nu_\mathrm{s}$ are the Poisson ratios and Young's moduli of tip and sample, respectively.

In the Hertz model, the adhesion of the sample is neglected, whereas two other theories do take the adhesion into account, the Johnson-Kendall-Roberts (JKR)[23] and Derjaguin-Müller-Toporov (DMT)[24] theories. The JKR theory can be applied in the case of large tips and soft samples with a large adhesion, the DMT theory in the case of small tips and stiff samples with a small adhesion. The used tip has an approximated radius of ≈ 20 nm and $VO_2$ is a stiff sample (see table 2). Therefore, the DMT theory is used.[19,20]

In the DMT theory, equation S1 is expanded to[24]

$$a = \sqrt[3]{RK(F + 2\pi RW)} \tag{S2}$$

with $W$ the work of adhesion per unit area. As the tip is approaching the surface, the two surfaces are attracted towards another. This introduces an additional force besides the force with which the tip presses on the surface (which is chosen by the user). An example of an $F(D_{\mathrm{PMC}})$ curve is shown in Fig. S8, in which $D_{\mathrm{PMC}}$ is the distance determined by the piezo motor controller. In order to get the displacement $D$, the information of the height sensor is also needed. The marked gray area in Fig. S8 is $W$.[20] The slope of the curve is constant over all the extension of the piezo motor controller, indicating a pure elastic, non-indentation model for the tip-sample contact.[25]

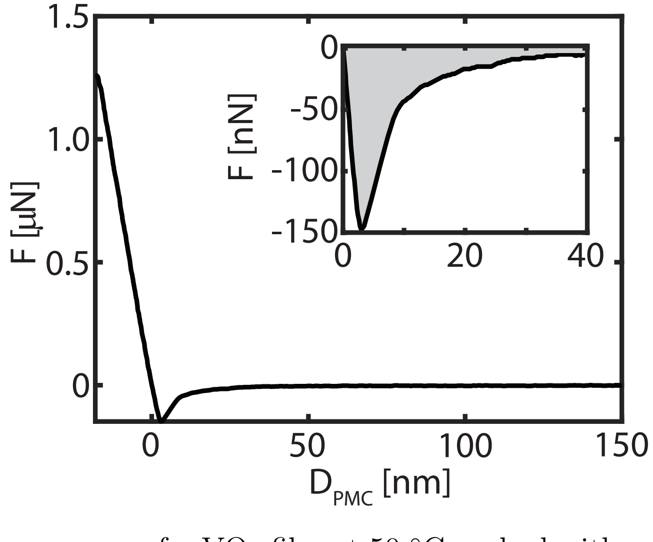


Figure S8: Force-distance curve of a $VO_2$ film at 50 °C probed with a boron-doped diamond tip with a tuned $k = 8\,\mathrm{N\,m^{-1}}$. The inset shows a zoom of the region near the origin. The gray marked region is defined as the work of adhesion per unit area, $W$.

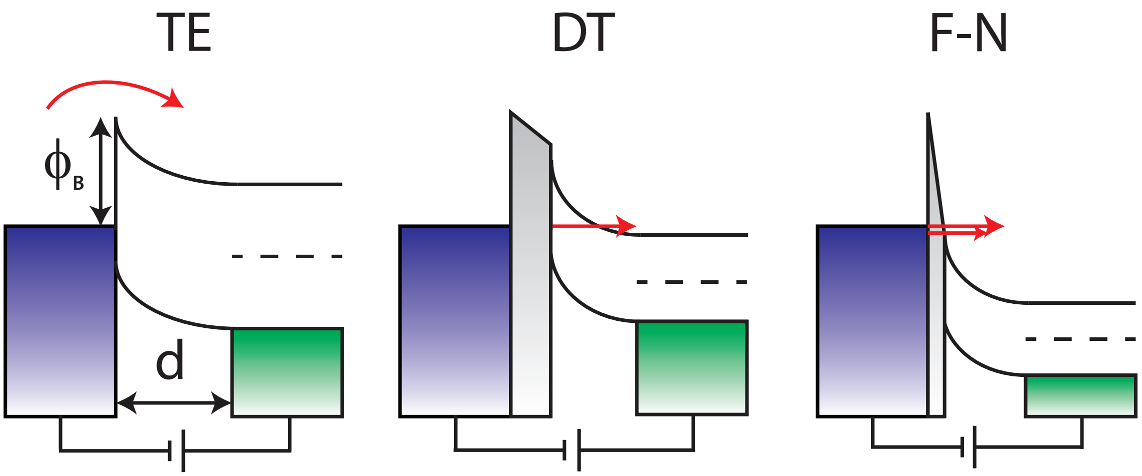


Figure S9: Schematic representation of the three injection mechanisms considered within the manuscript; thermionic emission (TE), direct tunneling (DT), and Fowler-Nordheim tunneling (F-N).

## 8 Charge injection mechanisms

From the measured $I(V)$ curves it is possible to acquire information about the Schottky barrier height ($\phi_\mathrm{B}$) and the dominant charge injection mechanism. The three different charge injection mechanisms are thermionic emission (TE), direct tunneling (DT) and Fowler-Nordheim tunneling (F-N). For TE, the Schottky barrier height and the ideality factor ($\eta$, this is the deviation of the charge transport from ideal thermionic emission) can be extracted. Within the TE model, the emission current $I$ is given by[26–28]

$$I = I_0 \exp\left(\frac{qV}{\eta k_\mathrm{B} T}\right)\left[1 - \exp\left(\frac{-qV}{k_\mathrm{B} T}\right)\right] \tag{S3}$$

where $V$ is the applied, $k_\mathrm{B}$ is the Boltzmann constant, $T$ the temperature, and $q$ is the electron charge. For values of $V$ larger than $3k_\mathrm{B}T/q$, the second term in equation S3 becomes negligible, equation S3 simplifies to:

$$I = I_0 \exp\left(\frac{qV}{\eta k_\mathrm{B} T}\right) \tag{S4}$$

The saturation current ($I_0$) is a constant and depends on $\phi_B$

$$I_0 = AA^*T^2 \exp\Big(-\frac{q\phi_B}{k_B T}\Big) \tag{S5}$$

with $A$ the effective contact area between the AFM tip and the $VO_2$, calculated using the formulas in section 7 and $A^*$ the Richardson constant ($A^* = \frac{4\pi q m^* k_B^2}{h^3}$, with $m^*$ the effective mass and $h$ the Planck constant). The ideality factor can be obtained from

$$\eta = \frac{q}{k_B T}\frac{dV}{d(\ln I)} \tag{S6}$$

and the Schottky barrier height ($\Phi_B$) is given by

$$\Phi_B = \frac{k_B T}{q}\ln\Big(\frac{A^* A T^2}{I_0}\Big) \tag{S7}$$

The ideality factor is extracted from the slope of the TE regime in the $\ln(I) - V$ plot, while the intercept of the curve gives the saturation current (see equation S5) which is used to extract $\phi_B$.

From F-N and DT, a product of the Schotty barrier height and the barrier width, referred to as the barrier parameter, can be obtained. It should be noted that the barrier parameter of F-N differs from the barrier parameter of DT. In a F-N plot, the F-N regime can be recognized as it satisfies the linear relation[28–30]

$$\ln\Big(\frac{I}{V^2}\Big) = \ln\Big(\frac{Aq^3 m_0}{8\pi h\phi d^2 m^*}\Big) - \frac{8\pi\sqrt{2m^*}\phi_B^{3/2} d}{3hqV} \tag{S8}$$

The slope of the F-N regime then equals $8\pi\sqrt{2m^*}\phi^{3/2}d/3hqV$, from which the barrier pa-

rameter $\phi^{3/2}d$ can be extracted. The barrier parameter of DT can also be obtained from the F-N plot. The DT regime satisfies[28–30]

$$\ln\left(\frac{I}{V^2}\right) = \ln\left(\frac{Aq^2\sqrt{2m^*\phi}}{Vh^2d}\right) - \frac{4\pi d\sqrt{2m^*\phi}}{h} \tag{S9}$$

Within the F-N plot, this regime can be plotted with a logarithmic function. The right term in equation S9 then equals $4\pi d\sqrt{2m^*\phi}/h$, from which the barrier parameter $\sqrt{\phi}d$ can be extracted. Because F-N is absent within our measurements, the DT parameters in the main text are extracted from a $\ln(I/V^2)$ versus $\ln(|1/V|)$ plot.

# 9 Error analysis

The uncertainty in the measurements is determined by calculating the standard deviation per measurement point in Fig. S10. Each measurement point in Fig. S10 consists of 64-144 measurements (see section 5). From each individual curve, the barrier height parameter is extracted, and based on these values, the standard deviation is determined. The standard deviation is visualized as the error bars in Fig. S10.

# 10 Thermionic emission data

An overview of the measurements performed on $VO_2$ (Fig. S11 and S12) and $TiO_2$ (Fig. S13) under varying pressures and temperatures. The results of the TE model are shown as well as the F-N plots.

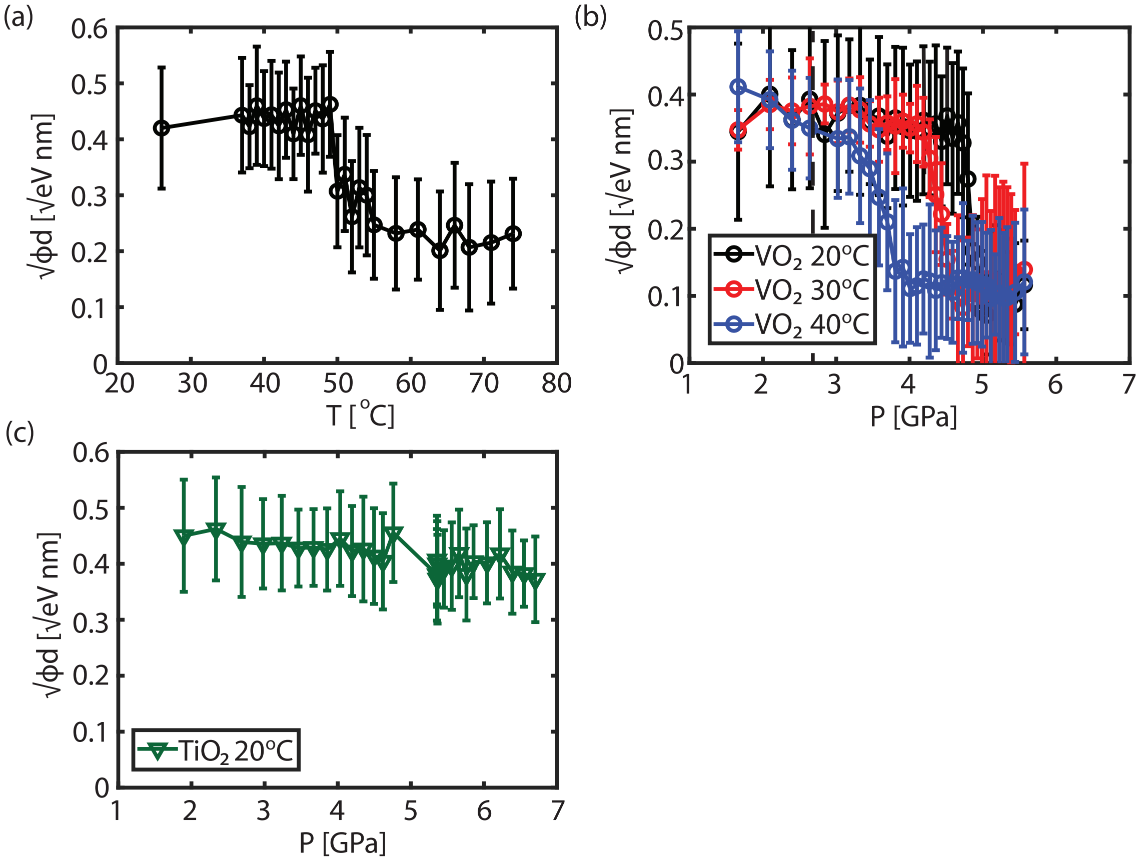


Figure S10: Figures 1D and 2B from the manuscript including error bars. (a) Fig. 1D of the manuscript. The obtained barrier height parameter versus the temperature for $VO_2$. (b) Fig. 2B of the manuscript. The obtained barrier height parameters versus pressure for $VO_2$ at different temperatures. (c) Fig. 2B of the manuscript. The barrier height parameter on $TiO_2$ versus pressure.

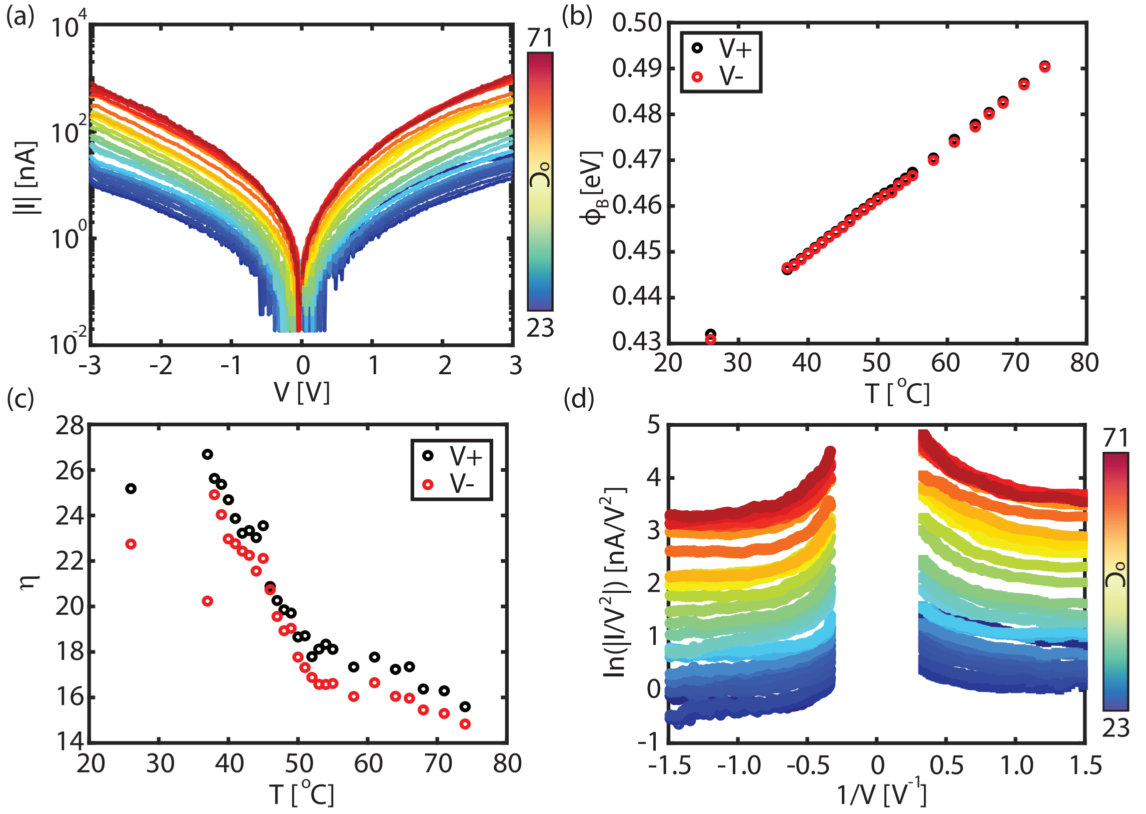


Figure S11: (a) The median $I(V)$ curves on $VO_2$ on a semi-logarithmic scale for different temperatures. (b) The extracted Schottky barrier height versus the temperature extracted using the thermionic emission model. A linear increase in $\phi_B$ with temperature is observed. (c) The obtained ideality factor as a function of temperature. With increasing temperature, the ideality factor decreases because the electrons gain more energy to cross the barrier. (d) F-N plots of the data in (a). Almost no linear relation is observed related to F-N tunneling. Therefore, the dominant transport mechanism is DT.

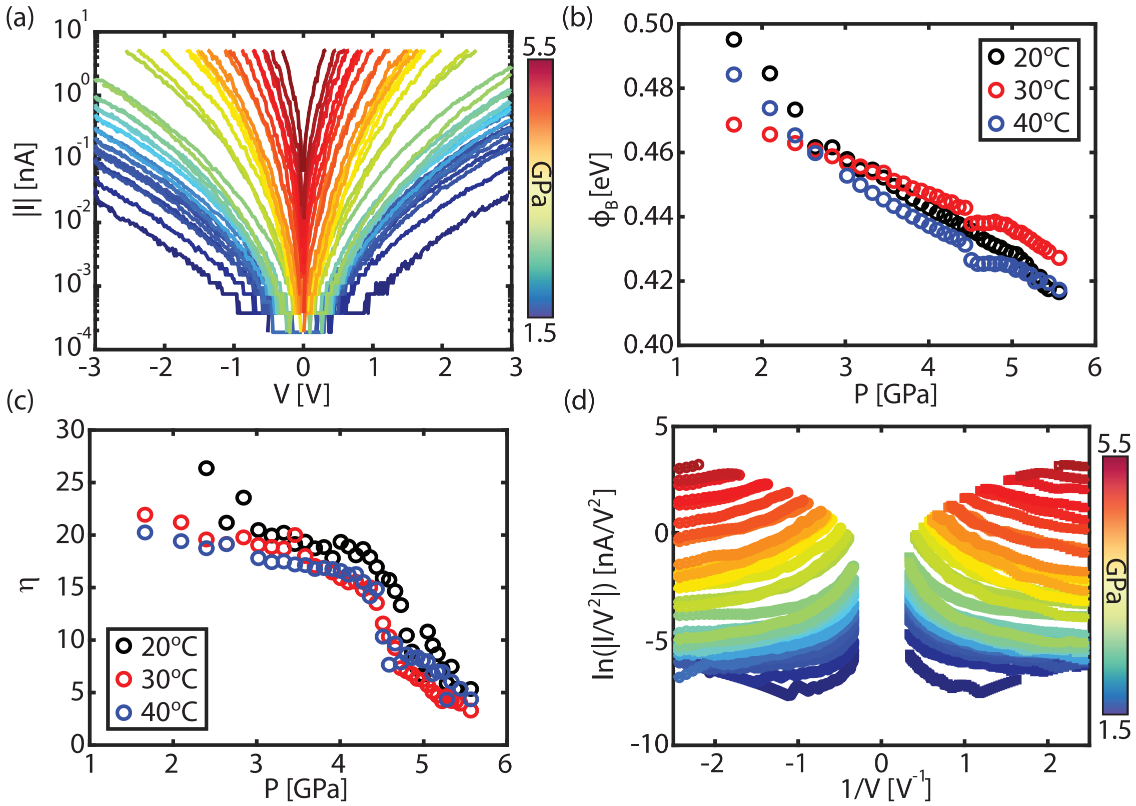


Figure S12: (a) The median $I(V)$ curves on $VO_2$ on a semi-logarithmic scale for different pressures at a temperature of 50 °C (b) The extracted Schottky barrier height as a function of pressure for three different temperatures. (c) The obtained ideality factor as a function of pressure for different temperatures. A change in slope is observed for all three temperatures at the same pressure. At higher pressures, the ideality factor drops to approx. 5. (d) (d) F-N plots of the data in (a). Almost no linear relation is observed related to F-N tunneling. Therefore, the dominant transport mechanism is DT.

.

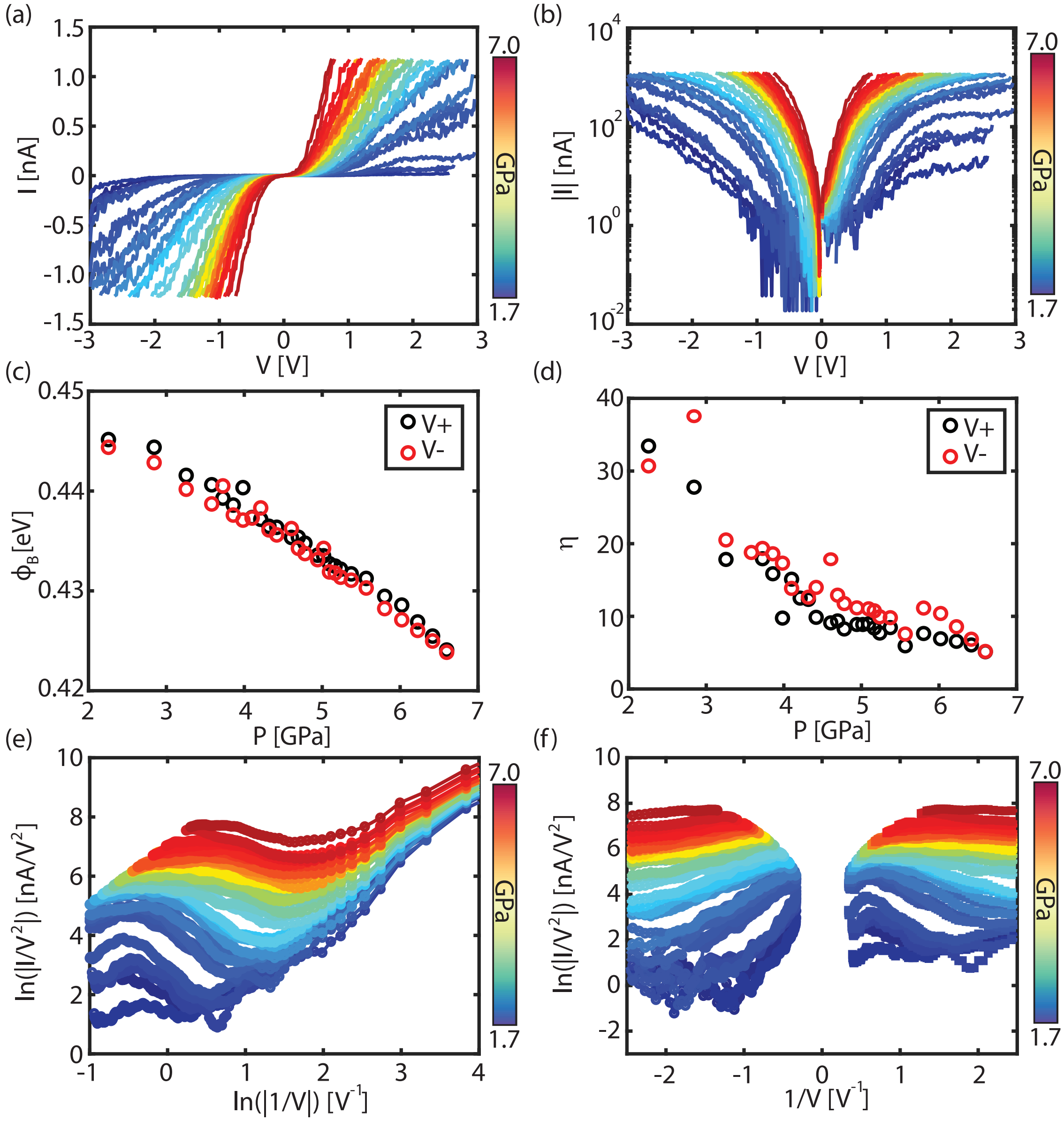


Figure S13: (a) The median $I(V)$ curves on $TiO_2$ for different pressures. (b) Same data as in (a) on a semi-logarithmic scale. (c-d) The extracted Schotty barrier height and ideality factor using the thermionic emission model (see equation S3) Similar to $VO_2$, thermionic emission is not the main charge injection mechanism ($\eta > 4$). (e) Same data as in (a) plotted on a $\ln(I/V^2)$ versus $\ln(|1/V|)$ scale (only the negative polarity is shown), which is related to the DT model. (f) F-N plots of the data in (a). Almost no linear relation is observed related to F-N tunneling. Therefore, the dominant transport mechanism is DT, very similar to $VO_2$.

# 11 X-ray photoemission spectroscopy

Soft and hard X-ray photoemission spectroscopy was performed *ex situ* using a dual beam PHI Quantes XPS/HAXPES scanning microprobe instrument equipped with monochromated Al (photon energy $E_{ph}^{Al} \approx 1486.7\,\mathrm{eV}$) and Cr anodes ($E_{ph}^{Cr} \approx 5414.8\,\mathrm{eV}$). By changing both the incident photon energy as well as the photoelectron takeoff angle (between the sample surface and the analyzer entrance), we can reconstruct an approximate depth profile of the sample. Maximum surface sensitivity is achieved for soft X-rays and shallow takeoff angles, here using the Al anode and a takeoff angle of 20°. Conversely, maximum bulk sensitivity is obtained for hard X-rays and normal emission, i.e., using the Cr anode and a takeoff angle of 90°. Following the analysis of Silversmit et al. [31], we measure the O 1s and V 2p features in the binding energy range from 550 - 505 eV, and reference all spectra to the O 1s peak at a binding energy of 530 eV. The diameter of the X-ray beam is set to 100 µm and the source is operated in the high power setting (100 W, 20 kV). A low-energy electron flood gun is used for charge neutralization and low energy Ar ions are used to remove the surplus of electrons put there by the electron flood gun. A working pressure of $1.3 \times 10^{-8}$ mbar is set. The base pressure of the vacuum system is better than $7 \times 10^{-9}$ mbar. The measurements are performed using a pass energy of 140 eV and a step size of 0.25 eV.

Furthermore, we observed that the $V^{5+}$ layer forms immediately and spontaneously, albeit less pronounced, even if the $VO_2$ sample is directly transferred in ultrahigh vacuum conditions from the growth to the X-ray photoemission chamber without intermittent exposure to the ambient air. This is displayed in Fig. S14. The *in situ* XPS data has been provided by courtesy of dr. Phu Le and has been previously published,[32] where it was erroneously assigned as $V^{4+}$ exclusively. This *in situ vs. ex situ* comparative study was performed with a takeoff angle of 90° and a monochromatic Al anode source (Omicron XM 1000).

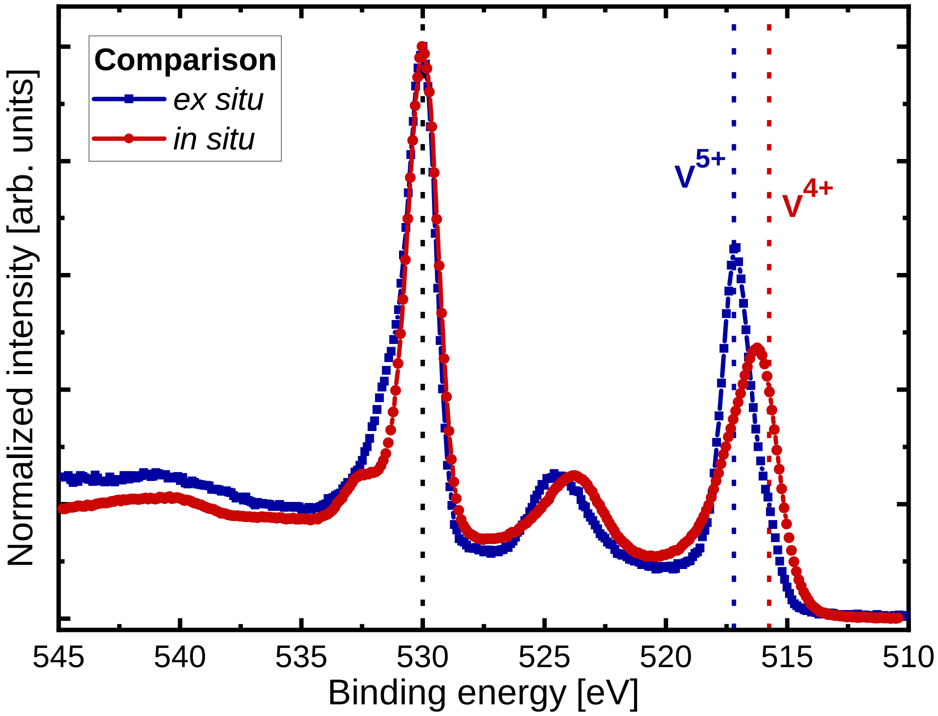


Figure S14: X-ray photoemission measurements of two $VO_2$ thin films, one transferred *in situ* to the analysis chamber, the other one *ex situ*, i.e. by exposing it to the ambient air. The ratio between $V^{4+}$ and $V^{5+}$ differs, where the surface of the *in situ* sample is less oxidized but is clearly not exclusively tetravalent. Both samples show a very sizable $V^{5+}$ contribution.

# 12 Scanning transmission electron microscopy

In addition to XRD and complementary to our XPS/HAXPES analysis, we also studied the $VO_2$ thin films by scanning transmission electron microscopy (STEM) and electron energy loss spectroscopy (EELS).

Due to the similar atomic masses of Ti and V, in high-angle annular dark-field (HAADF) imaging mode there is no clear contrast at the substrate-film interface. In Fig. 2 (d) in the main document, the atomic columns of Ti and V are virtually indiscernible, and therefore it is almost impossible to tell where the substrate ends and the film starts. This is why we turned to monochromatic EELS to investigate the interface spectroscopically, and found indeed a sharp interface with minimal interdiffusion, see the line profile in Fig. S15. To create this line profile, the intensities under the Ti and V L-edges were integrated, respectively. The obtained EEL spectra (see Fig. 2 (c) in the main document) are in excellent agreement with our earlier work and X-ray absorption spectroscopy.[33]

STEM-EELS was performed at the Electron Microscopy for Materials Science (EMAT) institute at the University of Antwerp in Belgium. Electron energy loss spectroscopy (EELS) data were acquired on a double aberration-corrected ThermoFischer Scientific Titan 80-300 electron microscope equipped with a Gatan K2 Summit camera installed at the end of a Quantum GIF. For these measurements, the microscope was operated at 120 kV in monochromated mode, providing an energy resolution of 120 meV with a beam current of 80 pA. A convergence angle of 19 mrad and a collection angle of 100 mrad were used for the acquisition. This low beam current was chosen to limit the beam-induced damage and the collection angle was maximized by the use of the EFSTEM camera lengths. An energy dispersion of 0.025 eV /pixel was used with an exposure time of 0.2 s /pixel at 100 % duty cycle of the K2 summit camera. A dose average over 32 pixels was used to further reduce beam damage. The HAADF STEM image shown in Fig. 2 (d) in the main document was acquired at 120 kV with a 20 mrad convergence angle and a collection angle of 70 mrad to 160 mrad.

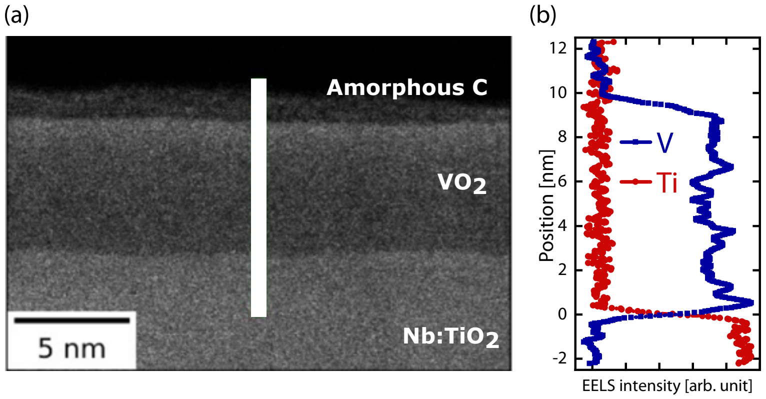


Figure S15: (a) Low magnification HAADF STEM image, (b) EELS intensity distribution for the Ti and V signals along the line indicated in (a).

# 13 Macroscopic transport measurements

In-plane four-point transport measurements in van der Pauw geometry reveal the well-known hysteresis curve for $VO_2$ upon heating and cooling across the phase transition temperature. Note that for this experiment, we grew 4 nm $VO_2$ film on an undoped rutile $TiO_2$ (001) substrate. Titanium-gold contacts were sputtered at the corners and connected via aluminum wire bonds to the puck of a cryogen-free Quantum Design Physical Properties Measurement System (PPMS DynaCool). In Fig. S16, we show the resistance as a function of temperature and observe an abrupt change of almost 3 decades across the MIT. Apart from the amplitude of the MIT, the two other key metrics are the sharpness of the transition described by a temperature window $\Delta T$ and the width of the thermal hysteresis $\Delta H$, which can both be calculated from Gaussian fits of the derivative of the resistance ($\frac{\mathrm{d}\log R}{\mathrm{d}T}$). We find a hysteresis $\Delta H = 9 \pm 1\,°\mathrm{C}$ defined as the difference in temperatures at which the derivative curves of the heating and cooling sweeps show their respective extrema, and a transition full width half maximum of $\Delta T = 5 \pm 1\,°\mathrm{C}$ for either sweep direction. According to Narayan and Bhosle[34] a narrow transition width is indicative of a low defect content, and the narrow hysteresis width points to a low concentration of low angle boundaries, both as expected for a coherently strained epitaxial film.

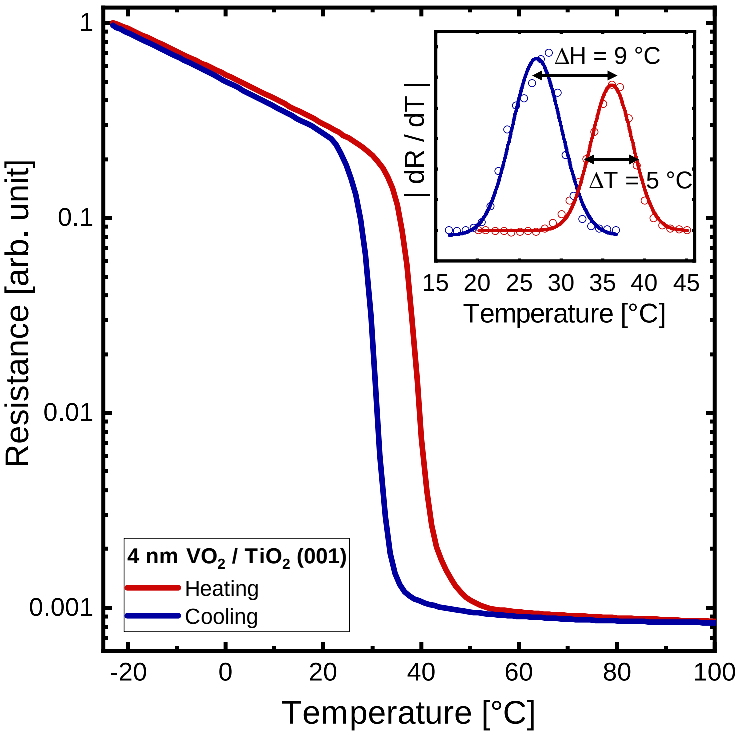


Figure S16: In-plane transport measurements as a function of temperature. Heating and cooling sweeps in red and blue, respectively. The inset shows the absolute value of the background-subtracted derivative dR/dT used to quantify the thermal hysteresis $\Delta H$ and transition sharpness $\Delta T$.

# References


(1) Krisponeit, J. O.; Fischer, S.; Esser, S.; Moshnyaga, V.; Schmidt, T.; Piper, L. F. J.; Flege, J. I.; Falta, J. The morphology of $VO_2/TiO_2(001)$: terraces, facets, and cracks. *Sci. Rep.* **2020**, *10*, 22374.

(2) Wahila, M. J.; Quackenbush, N. F.; Sadowski, J. T.; Krisponeit, J.-O.; Flege, J. I.; Tran, R.; Ong, S. P.; Schlueter, C.; Lee, T.-L.; Holtz, M. E.; Muller, D. A.; Paik, H.; Schlom, D. G.; Lee, W.-C.; Piper, L. F. J. The Breakdown of Mott Physics at $VO_2$ Surfaces. 2020.

(3) Stepanov, S. A. X-ray server: an online resource for simulations of X-ray diffraction and scattering. Advances in Computational Methods for X-Ray and Neutron Optics. 2004; pp 16 – 26.

(4) Hutter, J. L.; Bechhoefer, J. Calibration of atomic-force microscope tips. *Rev. Sci. Instrum.* **1993**, *64*, 1868–1873.

(5) Klein, C. A. Anisotropy of Young's modulus and Poisson's ratio in diamond. *Mater. Res. Bull.* **1992**, *27*, 1407–1414.

(6) Liu, K.; Zhang, B.; Wan, M.; Chu, J. H.; Johnston, C.; Roth, S. Measurement of electron affinity in boron-doped diamond from capacitance spectroscopy. *Appl. Phys. Lett.* **1997**, *70*, 2891–2893.

(7) Guo, H.; Chen, K.; Oh, Y.; Wang, K.; Dejoie, C.; Syed Asif, S. A.; Warren, O. L.; Shan, Z. W.; Wu, J.; Minor, A. M. Mechanics and dynamics of the strain-induced M1-M2 structural phase transition in individual $VO_2$ nanowires. *Nano Lett.* **2011**, *11*, 3207–13.

(8) Fan, W.; Huang, S.; Cao, J.; Ertekin, E.; Barrett, C.; Khanal, D. R.; Grossman, J. C.;

Wu, J. Superelastic metal-insulator phase transition in single-crystal $VO_2$ nanobeams. *Phys. Rev. B* **2009**, *80*.

(9) Sepúlveda, N.; Rúa, A.; Cabrera, R.; Fernández, F. Young's modulus of $VO_2$ thin films as a function of temperature including insulator-to-metal transition regime. *Appl. Phys. Lett.* **2008**, *92*, 191913.

(10) Tsai, K.-Y.; Chin, T.-S.; Shieh, H.-P. D. Effect of Grain Curvature on Nano-Indentation Measurements of Thin Films. *Jpn. J. Appl. Phys.* **2004**, *43*, 6268–6273.

(11) Aetukuri, N. B.; Gray, A. X.; Drouard, M.; Cossale, M.; Gao, L.; Reid, A. H.; Kukreja, R.; Ohldag, H.; Jenkins, C. A.; Arenholz, E.; Roche, K. P.; Dürr, H. A.; Samant, M. G.; Parkin, S. S. P. Control of the metal-insulator transition in vanadium dioxide by modifying orbital occupancy. *Nat. Phys.* **2013**, *9*, 661–666.

(12) Lee, S.; Meyer, T. L.; Sohn, C.; Lee, D.; Nichols, J.; Lee, D.; Seo, S. S. A.; Freeland, J. W.; Noh, T. W.; Lee, H. N. Electronic structure and insulating gap in epitaxial $VO_2$ polymorphs. *APL Mater.* **2015**, *3*, 126109.

(13) Yoon, H.; Choi, M.; Lim, T. W.; Kwon, H.; Ihm, K.; Kim, J. K.; Choi, S. Y.; Son, J. Reversible phase modulation and hydrogen storage in multivalent $VO_2$ epitaxial thin films. *Nat. Mater.* **2016**, *15*, 1113–9.

(14) Zhang, J.; Chen, S.; Jiang, Y.; Wang, C.; Qin, Z.; Qiu, X.; Yu, J.; Chen, Y.; Tang, Z. The photoelectron-imaging spectroscopic study and chemical bonding analysis of $VO_2$, $NbO_2$ and $TaO_2$. *RSC Adv.* **2020**, *10*, 41612–41617.

(15) Borgese, L.; Gelfi, M.; Bontempi, E.; Goudeau, P.; Geandier, G.; Thiaudière, D.; Depero, L. Young modulus and Poisson ratio measurements of $TiO_2$ thin films deposited with Atomic Layer Deposition. *Surf. Coat. Technol.* **2012**, *206*, 2459–2463.

(16) Pascual, J.; Camassel, J.; Mathieu, H. Fine structure in the intrinsic absorption edge of $TiO_2$. *Phys. Rev. B* **1978**, *18*, 5606–5614.

(17) Singh Surah, S.; Vishwakarma, M.; Kumar, R.; Nain, R.; Sirohi, S.; Kumar, G. Tuning the electronic band alignment properties of $TiO_2$ nanotubes by boron doping. *Results Phys.* **2019**, *12*, 1725–1731.

(18) Park, J. H.; Coy, J. M.; Kasirga, T. S.; Huang, C.; Fei, Z.; Hunter, S.; Cobden, D. H. Measurement of a solid-state triple point at the metal-insulator transition in $VO_2$. *Nature* **2013**, *500*, 431–434.

(19) Butt, H.-J.; Cappella, B.; Kappl, M. Force measurements with the atomic force microscope: Technique, interpretation and applications. *Surf. Sci. Rep.* **2005**, *59*, 1–152.

(20) Carpick, R. W.; Ogletree, D.; Salmeron, M. A General Equation for Fitting Contact Area and Friction vs Load Measurements. *J. Colloid Interface Sci.* **1999**, *211*, 395–400.

(21) Schrecongost, D.; Aziziha, M.; Zhang, H.-T.; Tung, I.-C.; Tessmer, J.; Dai, W.; Wang, Q.; Engel-Herbert, R.; Wen, H.; Picard, Y. N.; Cen, C. On-Demand Nanoscale Manipulations of Correlated Oxide Phases. *Adv. Func. Mater.* **2019**, *29*, 1905585.

(22) Hertz, H. Über die Berührung fester elastischer Körper. *J. für die Reine Angewandte Math.* **1881**, *92*, 156–171.

(23) Johnson, K. L.; Kendall, K.; Roberts, A. D. Surface energy and the contact of elastic solids. *Proc. R. Soc. Lond. A* **1971**, *324*, 401–313.

(24) Derjaguin, B.; Muller, V.; Toporov, Y. Effect of contact deformations on the adhesion of particles. *J. Colloid Interface Sci.* **1975**, *53*, 314–326.

(25) Domingo, N.; López-Mir, L.; Paradinas, M.; Holy, V.; Železný, J.; Yi, D.; Suresha, S. J.; Liu, J.; Rayan Serrao, C.; Ramesh, R.; Ocal, C.; Martí, X.; Catalan, G. Giant reversible

nanoscale piezoresistance at room temperature in $Sr_2IrO_4$ thin films. *Nanoscale* **2015**, *7*, 3453–3459.

(26) Roderick, E.; Williams, R. *Metal-Semiconductor Contacts*; Clarendon Press, Oxford, 1988.

(27) Sotthewes, K.; van Bremen, R.; Dollekamp, E.; Boulogne, T.; Nowakowski, K.; Kas, D.; Zandvliet, H. J. W.; Bampoulis, P. Universal Fermi-Level Pinning in Transition-Metal Dichalcogenides. *J. Phys. Chem. C* **2019**, *123*, 5411–5420.

(28) van Bremen, R.; Vonk, K.; Zandvliet, H. J. W.; Bampoulis, P. Environmentally Controlled Charge Carrier Injection Mechanisms of Metal/$WS_2$ Junctions. *J. Phys. Chem. Lett.* **2019**, *10*, 2578–2584.

(29) Beebe, J. M.; Kim, B.; Gadzuk, J. W.; Frisbie, C.; Kushmerick, J. G. Transition from Direct Tunneling to Field Emission in Metal-Molecule-Metal Junctions. *Phys. Rev. Lett.* **2006**, *97*, 026801.

(30) Ikuno, T.; Okamoto, H.; Sugiyama, Y.; Nakano, H.; Yamada, F.; Kamiya, I. Electron transport properties of Si nanosheets: Transition from direct tunneling to Fowler-Nordheim tunneling. *Appl. Phys. Lett.* **2011**, *99*, 023107.

(31) Silversmit, G.; Depla, D.; Poelman, H.; Marin, G. B.; De Gryse, R. Determination of the V2p XPS binding energies for different vanadium oxidation states ($V^{5+}$ to $V^{0+}$). *J. Electron Spectrosc. Relat. Phenom.* **2004**, *135*, 167–175.

(32) Le, P. T. P.; Huang, S.; Nguyen, M. D.; ten Elshof, J. E.; Koster, G. Tuning the metal insulator transition of vanadium dioxide on oxide nanosheets. *Appl. Phys. Lett.* **2021**, *119*.

(33) Le, P. T. P.; Hofhuis, K.; Rana, A.; Huijben, M.; Hilgenkamp, H.; Rijnders, G.; ten Elshof, J. E.; Koster, G.; Gauquelin, N.; Lumbeeck, G.; Schüßler-Langeheine, C.;

Popescu, H.; Fortuna, F.; Smit, S.; Verbeek, X. H.; Araizi-Kanoutas, G.; Mishra, S.; Vaskivskyi, I.; Dürr, H. A.; Golden, M. S. Tailoring Vanadium Dioxide Film Orientation Using Nanosheets: a Combined Microscopy, Diffraction, Transport, and Soft X-Ray in Transmission Study. *Adv. Funct. Mater.* **2019**, *30*, 1900028.

(34) Narayan, J.; Bhosle, V. M. Phase transition and critical issues in structure-property correlations of vanadium oxide. *J. Appl. Phys.* **2006**, *100*.
